# Rhodium-Catalyzed Arene Alkenylation Using Benzoquinone Derivatives as Oxidants

**DOI:** 10.1021/acs.organomet.5c00500

**Published:** 2026-02-10

**Authors:** Marc T. Bennett, Marina Goupalova, Christopher M. Chapman, Diane A. Dickie, T. Brent Gunnoe

**Affiliations:** Department of Chemistry, 2358 University of Virginia, Charlottesville, Virginia 22904, United States

## Abstract

The Rh-catalyzed conversion of olefins and arenes to alkenyl arenes using [(η^2^-C_2_H_4_)_2_Rh­(μ-OPiv)]_2_ as the catalyst precursor and 12 ortho- and para-substituted benzoquinone derivatives as the in situ oxidant is reported. Included are comparative studies of the quinone derivatives for (1) rate of styrene production from benzene and ethylene, (2) Markovnikov to anti-Markovnikov selectivity for reactions of benzene and propylene, and (3) ortho/meta/para selectivity when using *tert*-butylbenzene as the arene. Cyclic voltammetry was utilized to measure reduction potentials for each quinone to determine any possible influence of the quinone redox potential on arene alkenylation rate and selectivity. While significant differences in selectivity are observed between *ortho*-quinone derivatives, such differences are minimal when para-substituted quinones are utilized. These results suggest that *ortho*-benzoquinone derivatives likely serve as bidentate ligands, which explains the stronger influence on catalyst activity of *ortho*-benzoquinone identity compared to *para*-benzoquinones. Although *ortho*-benzoquinones generally give styrene production rates faster than those of *para*-benzoquinones, 3,5-di-*tert*-butyl-*ortho*-benzoquinone and *ortho*-chloranil react with ethylene to form bicyclo[2.2.2]­oct-5-ene-2,3-dione derivatives as a significant side product.

## Graphical abstract

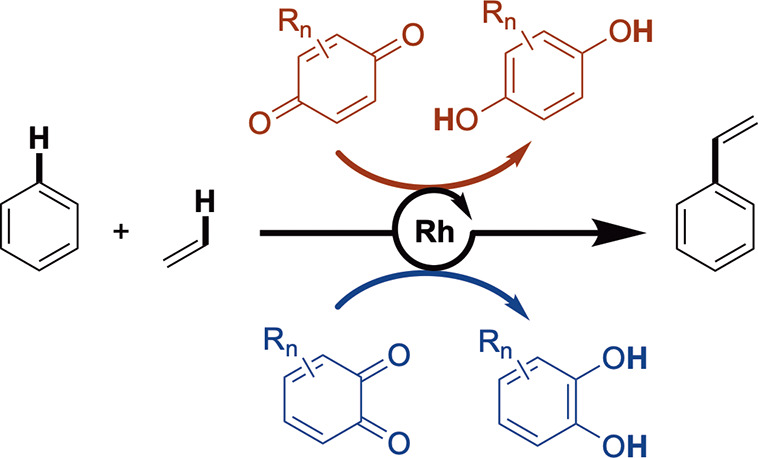

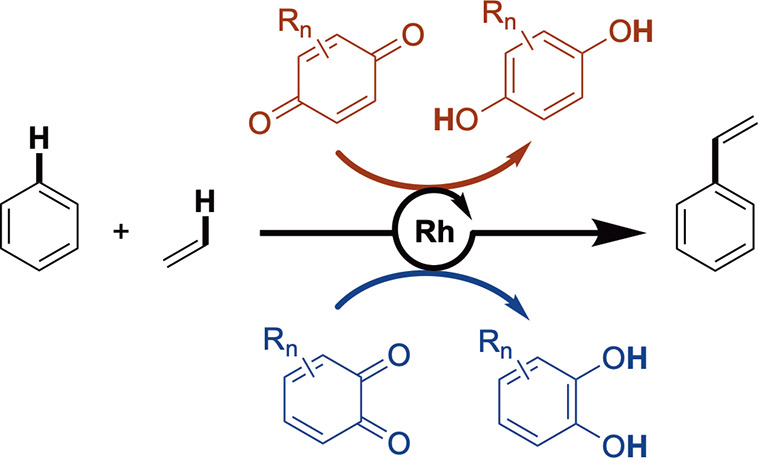

## Introduction

Alkyl and alkenyl arenes are used as precursors to polymers, fragrances, agricultural products, and pharmaceuticals.^1,2^ Styrene, for example, is produced on a large scale and is commonly synthesized by an energy-intensive ethylbenzene dehydrogenation process.^3,4^ The synthesis of ethylbenzene from ethylene and benzene operates through an acid-catalyzed mechanism involving ethylene protonation and electrophilic aromatic substitution of the formed ethyl cation with benzene.^4–9^ Ethylbenzene is more electron-rich than benzene and, hence, undergoes electrophilic aromatic substitution more rapidly than benzene. Accordingly, even at low benzene conversion, substantial quantities of polyethylbenzene side products are obtained. To improve ethylbenzene yield, industrial processes often incorporate a transalkylation step to convert undesired polyethylbenzenes to ethylbenzene.^8^

The direct oxidative conversion of arenes and olefins to alkenyl arenes (Scheme 1) offers potential advantages compared to arene alkylation followed by dehydrogenation.^10–12^ Our group and others have reported Rh-,^13–18^ Pd-,^19–21^ Ru-,^22,23^ and Ir-catalyzed^24^ arene alkenylation reactions. These catalysts are proposed to operate through transition metal-mediated arene C–H activation, olefin insertion into the formed M–Ar (Ar = aryl) bond, β-hydride elimination, and oxidation of a M–H intermediate by an in situ oxidant. These catalysts operate through similar mechanisms to Ir,^25–29^ Ru,^30–35^ Pt,^36–41^ and Ni^42,43^ catalysts for the conversion of olefins and arenes to alkyl arenes. Catalysis with molecular Rh, Pd, and Ir catalysts using dioxygen-recyclable Cu­(II)^17,19,24,44^ or Fe­(III)^13^ carboxylates as direct oxidants has been reported. Also, dioxygen can serve as the sole oxidant for Rh-catalyzed arene alkenylation, although turnover frequency (TOF) and selectivity are significantly decreased relative to catalysis using air recyclable Cu­(II) or Fe­(III) carboxylates in the absence of dioxygen during catalysis.^15,18,45,46^ While optimal catalytic activity is observed using Cu­(II) carboxylate salts as the oxidant, Cu­(II) carboxylates mediate stoichiometric formation of phenyl esters.^45,47^ Identification of oxidants that are dioxygen-recyclable and that do not undergo undesired side reactions is critical for improving reaction rate and selectivity.

**Scheme 1:**
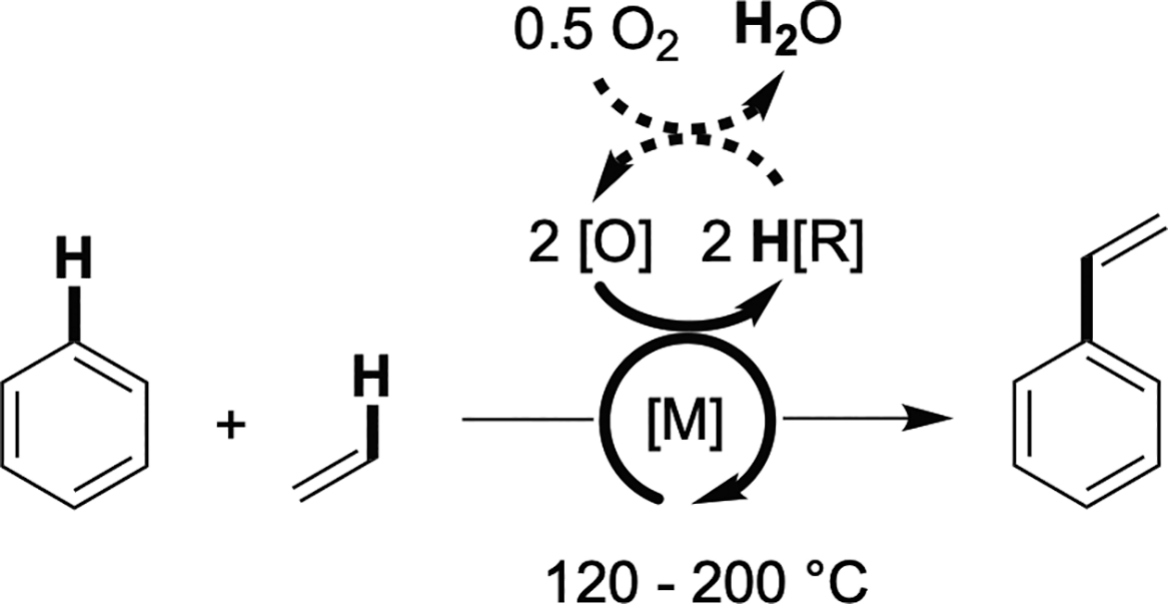
General Reaction Scheme for Transition Metal-Catalyzed Arene Alkenylation^a^

a [O] represents an in situ oxidant, H­[R] represents the reduced form of the in situ oxidant, and [M] represents the transition metal catalyst, typically a Rh, Pd, Ir, or Ru complex. Dashed lines indicate possible reoxidation of [R] to [O] using dioxygen, which can be performed for reduced forms of Cu- and Fe-based oxidants.

Previously, our group observed significant variation in Rh catalyst activity and selectivity as a function of oxidant identity.^18^ We probed the use of dioxygen alone as the oxidant in addition to Fe­(III) and Cu­(II) carboxylates in both the presence and absence of dioxygen. The kinetics of styrene production followed the trend Cu­(II) > Fe­(III) > O_2_, and the selectivity of benzene propenylation and monosubstituted arene ethenylation varied substantially as a function of oxidant identity. While these trends could be effects directly related to oxidant strength (e.g., Rh–H oxidation kinetics and undesired catalyst oxidation/reduction), catalyst speciation is also likely influenced by oxidant identity. For example, it was found that under certain conditions, Cu­(II) carboxylates react with catalyst precursor [(η^2^-C_2_H_4_)_2_Rh­(μ-OAc)]_2_ to form the heterotrinuclear species [(η^2^-C_2_H_4_)_2_Rh­(μ-OPiv)]_2_(μ-Cu).^20^ Further studies suggested that this Rh_2_Cu complex likely exists in a complicated equilibrium with RhCu complexes.^19^ Similarly, we reported that when using Pd­(OAc)_2_ as the catalyst precursor, conversion to heterotrimetallic PdCu_2_(μ-OPiv)_6_(η^2^-C_2_H_4_)_3_, which is likely in equilibrium with Pd_2_Cu complexes, occurs.^19,20^ Since oxidant identity can influence catalyst speciation, it is challenging to deduce whether changes in selectivity and reaction rate are the result of catalyst structure or are direct effects of oxidant strength (e.g., Rh–H oxidation kinetics or catalyst oxidation). Also, the scope of Fe­(III)- and Cu­(II)-based oxidants that are soluble in benzene is limited; therefore, a systematic study of the effect of oxidant strength on catalyst activity and selectivity is not straightforward. To better understand the influence of the oxidant redox potential on catalyst selectivity and activity, we sought out a structurally and electronically tunable oxidant.

Benzoquinone and its derivatives are common additives for hydrocarbon oxidative functionalization reactions catalyzed by late transition metals. Such reactions include Wacker oxidation,^48^ olefin–arene oxidative coupling,^49,50^ arene–arene oxidative coupling,^51^ and olefin acetoxylation.^52^ In some cases, benzoquinone serves as the species that oxidizes a transition metal intermediate (Scheme 2). This includes (1) use of stoichiometric benzoquinone as the sole oxidant,^49,53,54^ (2) use of a cocatalyst to mediate hydroquinone reoxidation by dioxygen,^55–60^ and (3) use of dioxygen^61^ or another stoichiometric oxidant^62^ to reoxidize substoichiometric hydroquinone. In other cases, *para*-benzoquinone has been proposed to mediate mechanistic steps not related to catalyst oxidation by η^2^-binding to a transition metal catalyst.^51,63^ Importantly, some hydroquinone derivatives can be reoxidized to quinones using dioxygen, although a catalyst is typically necessary to facilitate oxidation.^64–66^

**Scheme 2:**
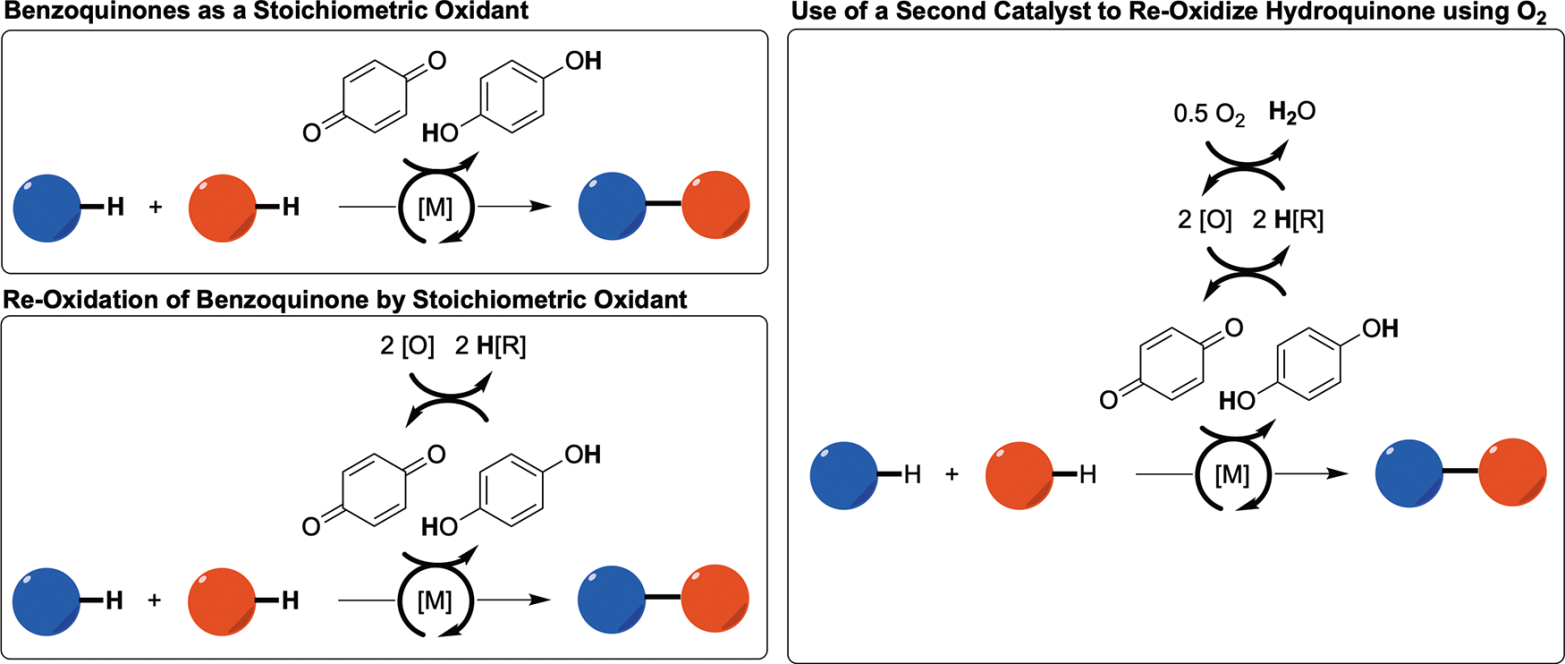
Generic Representations of Previously Reported Uses of Benzoquinone and Its Derivatives as an Oxidant or Co-oxidant for Transition Metal-Catalyzed Hydrocarbon Oxidative Functionalization Reactions

To better understand the role of the oxidant structure and oxidizing ability on the rate and selectivity of Rh-catalyzed arene alkenylation, we sought an oxidant that is structurally and electronically modulable. Given the precedent of benzoquinone derivatives serving as oxidants for late-transition-metal-catalyzed oxidative functionalization processes, we speculated that benzoquinone and its derivatives might be suitable oxidants for Rh-catalyzed arene alkenylation. Herein, we report Rh-catalyzed arene alkenylation by comparing the use of 12 *ortho*- and *para*-benzoquinone derivatives as the in situ oxidant under anaerobic conditions. Comparisons include the rate of styrene production from benzene and ethylene, selectivity for Markovnikov versus anti-Markovnikov products when using propylene as the olefin, and ortho/meta/para selectivity when *tert*-butylbenzene was used as the arene.

## Results and Discussion

### Identification of Reaction Conditions

With the goal of identifying reaction conditions for which Rh-catalyzed benzene ethenylation can occur with benzoquinone-based oxidants, we performed initial studies using 3,5-di-*tert*-butyl-*ortho*-benzoquinone (BQ) as the in situ oxidant (Figure 1). Reaction conditions were analogous to those reported previously using Fe and Cu carboxylates as oxidants.^13,18^ In neat benzene, 0.001 mol % (relative to benzene per single Rh atom) of [(η^2^-C_2_H_4_)_2_Rh­(μ-OPiv)]_2_ was combined with 240 equiv (relative to single Rh atom) of 3,5-di-*tert*-butyl-*ortho*-benzoquinone, 960 equiv of HOPiv, and 70 psig of ethylene at a reaction temperature of 170 °C in the absence of air. Under these conditions, 86(23) turnovers (TOs) of styrene were produced after 2 h. Also, 46(10) equiv of side product 1,5-di-*tert*-butylbicyclo­[2.2.2]­oct-5-ene-2,3-dione, which also forms in the absence of Rh (see the Experimental Section for the synthesis of 1,5-di-*tert*-butylbicyclo­[2.2.2]­oct-5-ene-2,3-dione in the absence of Rh), was observed upon reaction of 3,5-di-*tert*-butyl-*ortho*-benzoquinone with ethylene. Attempted use of 240 equiv of 1,5-di-*tert*-butylbicyclo­[2.2.2]­oct-5-ene-2,3-dione as the in situ oxidant in place of 3,5-di-*tert*-butyl-*ortho*-benzoquinone resulted in no styrene formation after 2 h. A reaction performed with 960 equiv of KOPiv in place of HOPiv resulted in 10(2) TOs of styrene. The use of 10 equiv of 3,5-di-*tert*-butyl-*ortho*-benzoquinone results in the production of 7.0(3) TOs of styrene (∼70% yield based on 3,5-di-*tert*-butyl-*ortho*-benzoquinone as the limiting reagent), consistent with one equiv of 3,5-di-*tert*-butyl-*ortho*-benzoquinone being consumed per each TO of styrene. No styrene was observed for reactions lacking any one component: [(η^2^-C_2_H_4_)_2_Rh­(μ-OPiv)]_2_, HOPiv, or 3,5-di-*tert*-butyl-*ortho*-benzoquinone.

**Figure 1:**
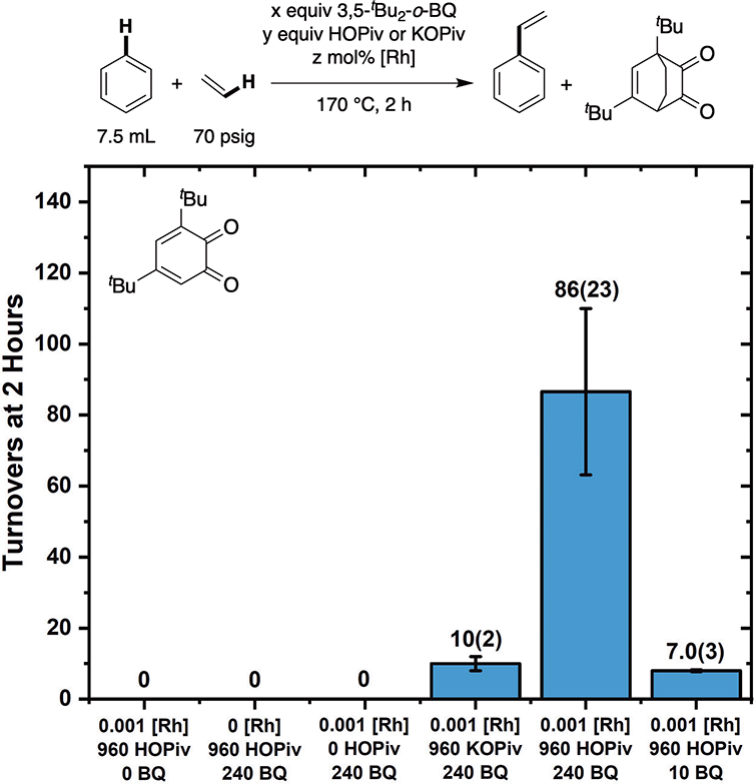
Studies on the effect of 3,5-di-*tert*-butyl-*ortho*-benzoquinone (BQ), HOPiv, or KOPiv and [(η^2^-C_2_H_4_)_2_Rh­(μ-OPiv)]_2_ being present under reaction conditions. Reaction conditions: 7.5 mL benzene, 0.001 or 0 mol % (relative to benzene per single Rh atom) [(η^2^-C_2_H_4_)_2_Rh­(μ-OPiv)]_2_, 240, 10, or 0 equiv (relative to Rh) 3,5-di-*tert*-butyl-*ortho*-benzoquinone, 960 or 0 equiv HOPiv or KOPiv, 70 psig ethylene, 170 °C. Each data point represents the average of a minimum of three independent experiments, and the error bars represent the standard deviation from the multiple experiments.

Having identified reaction conditions for which a benzoquinone derivative is an active oxidant for Rh-catalyzed benzene ethenylation, we studied the kinetics of styrene production as a function of the benzoquinone identity. As shown in Figure 2 (left side), among the *ortho*-benzoquinone derivatives, the rate of styrene production, determined by using the initial number of turnovers (TOs) at the 2 h time point, follows the trend 1,2-naphthoquinone > 3,5-di-*tert*-butyl-*ortho*-benzoquinone > 9,10-phenanthrene quinone > *ortho*-chloranil. The rates of reaction using substituted *para*-benzoquinones are generally slower than those observed with *ortho*-benzoquinones (Figure 2, right side), with *para*-chloranil and 2,5-dichloro-*para*-benzoquinone giving the fastest catalysis. Among para-substituted quinones, 2-chloro-*para*-benzoquinone gives the next fastest rate of reaction, followed by *para*-benzoquinone and *para*-fluoranil. Minimal reactivity was observed for 2,5-di-*tert*-butyl-*para*-benzoquinone, tetramethyl-*para*-benzoquinone, and anthraquinone. As noted above, 3,5-di-*tert*-butyl-*ortho*-benzoquinone undergoes a Diels–Alder reaction with ethylene to form 1,5-di-*tert*-butylbicyclo­[2.2.2]­oct-5-ene-2,3-dione. When *ortho*-chloranil was used as the oxidant, a product consistent with 1,4,5,6-tetrachlorobicyclo[2.2.2]­oct-5-ene-2,3-dione was observed but not quantified by gas chromatography-mass spectrometry (GC-MS) (Figure S24). Analogous products were not observed using 9,10-phenanthrene dione or naphthoquinone.

**Figure 2:**
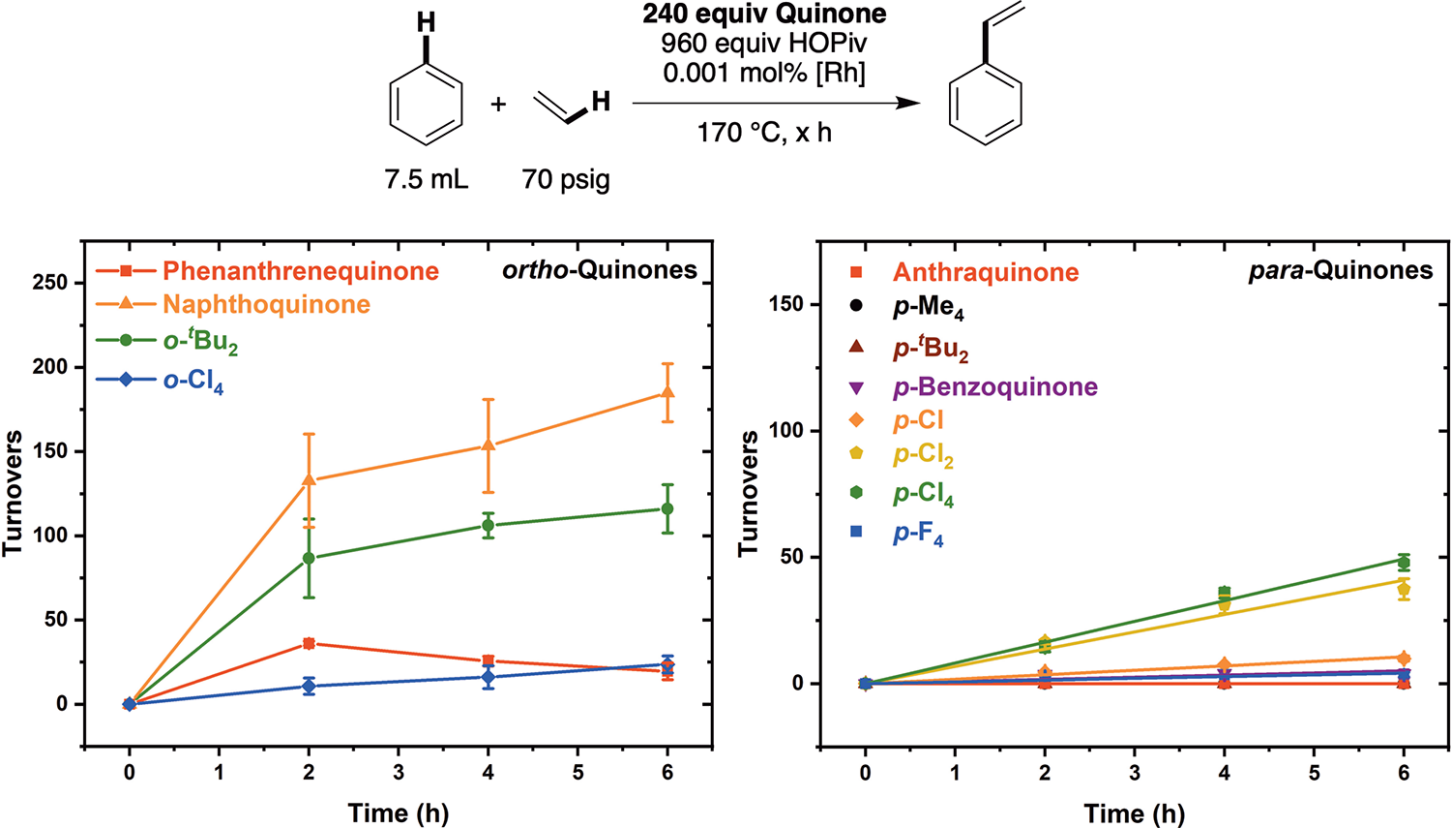
Kinetics of benzene ethenylation as a function of the functionalized *ortho*- or *para*-benzoquinone identity. Reaction conditions: 7.5 mL of benzene, 0.001 mol % (relative to benzene per single Rh atom) [(η^2^-C_2_H_4_)_2_Rh­(μ-OPiv)]_2_, 240 equiv (relative to Rh) benzoquinone, 960 equiv HOPiv, 70 psig ethylene, and 170 °C. Each data point represents the average of a minimum of three independent experiments, and the error bars represent the standard deviation from the multiple experiments.

To quantify the influence of quinone oxidizing ability on the rate of benzene ethenylation, we performed cyclic voltammetry to measure quinone reduction potentials using ferrocene as an internal standard (see the Supporting Information for more details). Reduction potentials for these benzoquinone derivatives have been reported previously,^67–69^ but we repeated measurements to ensure identical conditions for each quinone. Turnovers of styrene produced after 2 h of reaction were plotted as a function of benzoquinone reduction potential to approximate the relationship between benzoquinone reduction potential and turnover frequency (TOF) (Figure 3). For the *para*-benzoquinone substrates, styrene production TOF generally increases as the quinone oxidizing ability increases. Despite having a reduction potential similar to that of *para*-chloranil, minimal reactivity was observed using *para*-fluoranil, perhaps indicating a catalyst deactivation pathway with *para*-fluoranil. With *ortho*-quinones, for which four substrates were probed, the TOF increases from the weakest oxidant, phenanthrene dione, to naphthoquinone. Use of the strongest oxidant among the *ortho*-quinones, *ortho*-chloranil, results in the slowest reaction rate among the *ortho*-quinones. Excluding *ortho*-chloranil, the use of *ortho*-benzoquinone-based oxidants results in reaction rates significantly faster than those achieved using *para*-benzoquinones. Cyclic voltammograms were obtained for the four *ortho*-benzoquinone derivatives in the presence of HOPiv to quantify the influence of HOPiv on the quinone oxidizing ability. The addition of 1–4 equiv of HOPiv results in the observation of a single redox event for all four of the benzoquinone derivatives, which is consistent with two-electron redox chemistry occurring in the presence of HOPiv (Figures S18–S21). As shown in Table S3, the *E*_1/2_ for naphthoquinone is more negative than that of 3,5-di-*tert*-butyl-*ortho*-benzoquinone, while the first redox event observed in the absence of HOPiv follows the reverse trend. Nevertheless, plotting TOF as a function of *E*_1/2_ values obtained in the presence of HOPiv yields a similarly complicated dependence on *E*_1/2_ compared to the *E*_1/2_ values measured in the absence of HOPiv (Figure S22).

**Figure 3:**
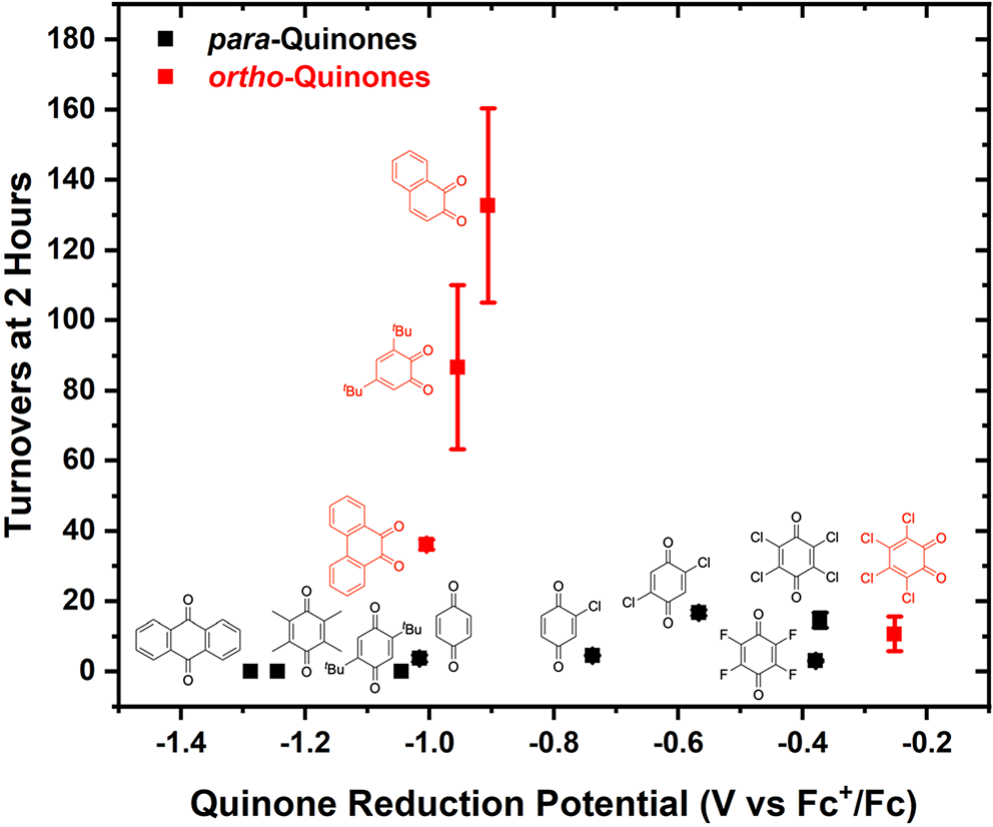
Benzene ethenylation turnover frequency (represented by TOs of styrene measured after 2 h) versus the reduction potential for benzoquinone derivatives’ first reduction. Cyclic voltammograms were recorded in degassed MeCN with 100 mM [*N*-Bu_4_]­[PF_6_] (*N*-Bu_4_ = tetrabutylammonium) as the supporting electrolyte, and reduction potentials are referenced to ferrocene, which was used as an internal standard. Working electrode: glassy carbon; counter electrode: Pt wire; reference electrode: Ag/AgNO_3_. Each data point for styrene turnovers at 2 h represents the average of a minimum of three independent experiments, and the error bars represent the standard deviation from multiple experiments.

For the catalytic alkenylation of benzene using propylene, four primary products are observed: allylbenzene, β-*cis*-methylstyrene, β-*trans*-methylstyrene, and α-methylstyrene. Three of these products, allylbenzene, β-*cis*-methylstyrene, and β-*trans*-methylstyrene, result from anti-Markovnikov selectivity (linear selectivity), while α-methylstyrene results from Markovnikov selectivity (branched selectivity). Given the influence of quinone identity on the reaction rate, we speculated that quinone identity might also modulate linear/branched selectivity when propylene is used as the olefin. Previously, we studied linear/branched selectivity using dioxygen, Cu­(II) carboxylates, and Fe­(III) carboxylates as oxidants, and we observed substantial changes in selectivity as the oxidant was varied.^18^ When using Cu­(II) carboxylates as oxidants, we have proposed that Cu­(II) is likely embedded in the active catalyst(s).^14,19^ Thus, the change in linear/branched selectivity as a function of oxidant likely is due, at least in part, to a change in the identity of the active catalyst(s). However, it is unclear whether the variation in selectivity is solely attributable to the active catalyst structure or if changes in the reaction pathway, as a result of differences in Rh–H oxidation kinetics, also contribute.

As shown in Figure 4, using *ortho*-benzoquinones, the linear/branched selectivity ranges from 2.6(1):1 to 4.8(1):1. With *para*-benzoquinone substrates, linear/branched selectivity does not follow any clear trend as a function of quinone identity and remains between 2:1 and 3:1 (linear/branched) in most cases (Figure 5).

**Figure 4:**
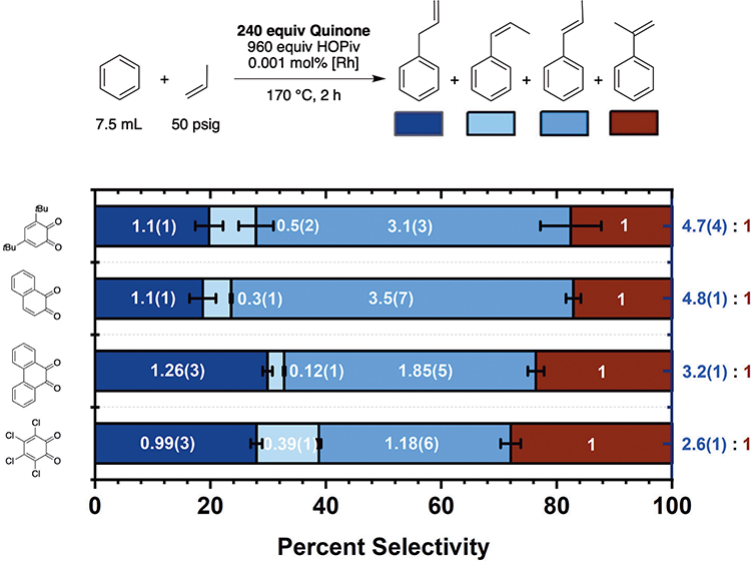
Selectivity of benzene propenylation as a function of the *ortho*-benzoquinone identity. Reaction conditions: 7.5 mL benzene, 0.001 mol % (relative to benzene per single Rh atom) [(η^2^-C_2_H_4_)_2_Rh­(μ-OPiv)]_2_, 240 equiv (relative to Rh) benzoquinone, 960 equiv HOPiv, 50 psig propylene, 170 °C, 2 h. Each data point represents the average of a minimum of three independent experiments, and the error bars represent the standard deviation from multiple experiments.

**Figure 5:**
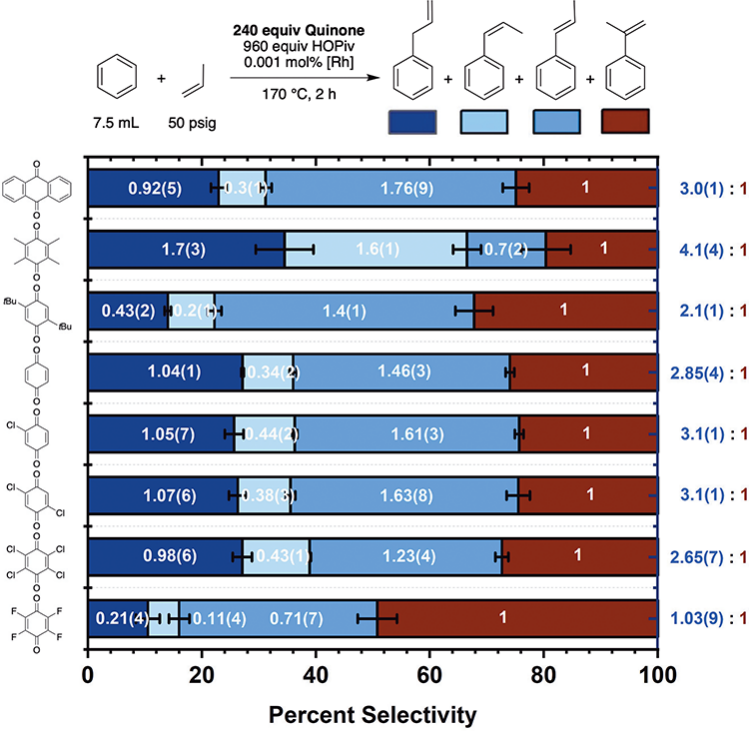
Selectivity of benzene propenylation as a function of the *para*-benzoquinone identity. Reaction conditions: 7.5 mL of benzene, 0.001 mol % (relative to benzene per single Rh atom) [(η^2^-C_2_H_4_)_2_Rh­(μ-OPiv)]_2_, 240 equiv (relative to Rh) benzoquinone, 960 equiv HOPiv, 50 psig propylene, 170 °C, 2 h. Each data point represents the average of a minimum of three independent experiments, and the error bars represent the standard deviation from multiple experiments.

To determine if quinone oxidizing ability influences linear/branched selectivity, we plotted linear/branched selectivity as a function of the quinone reduction potential. For both *para-*quinones and *ortho*-quinones, no clear trend is observed as a function of redox potentials measured in the absence of HOPiv (Figure 6). Likewise, plotting linear/branched selectivity as a function of *E*_1/2_ values for *ortho*-quinones measured in the presence of HOPiv results in a trend similar to that of *E*_1/2_ measured in the absence of HOPiv (Figure S23).

**Figure 6:**
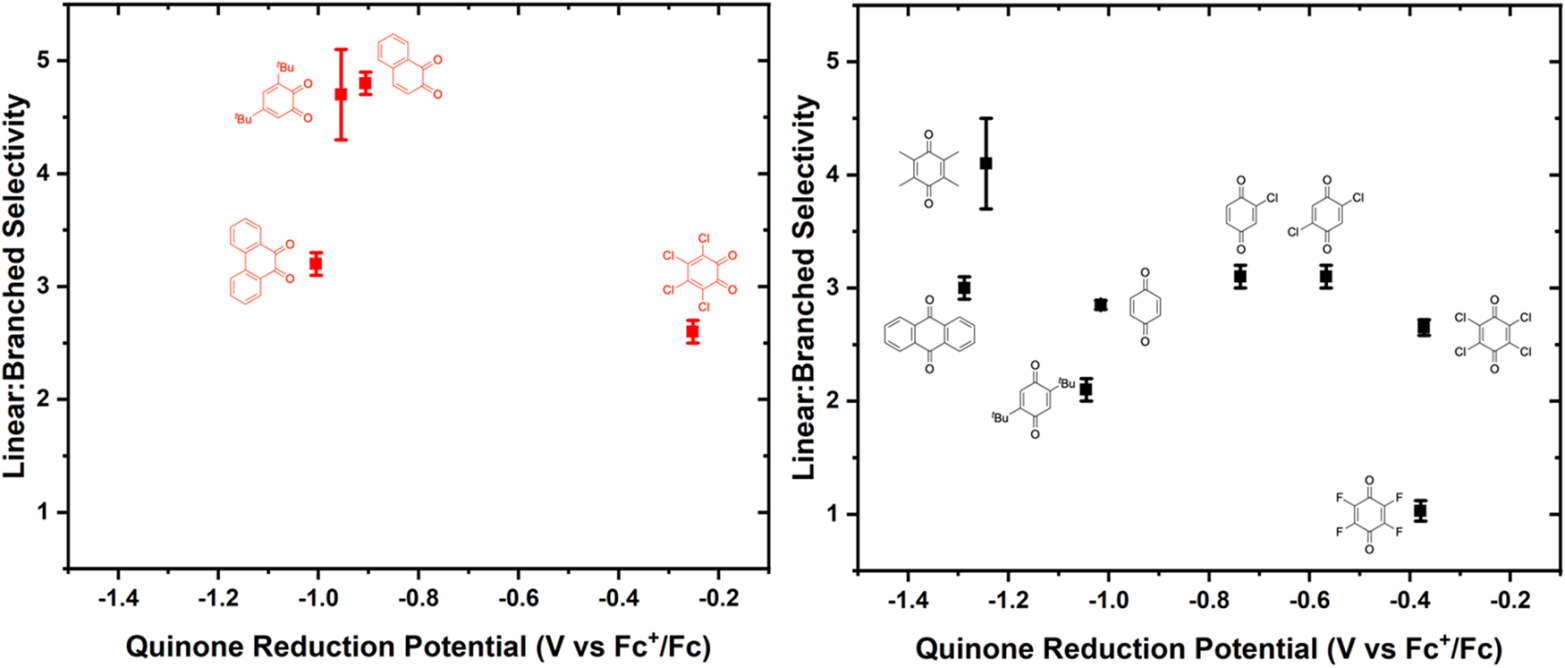
Linear/branched selectivity for benzene propenylation as a function of benzoquinone derivative reduction potential for *ortho*- and *para*-quinones. Reaction conditions: 7.5 mL benzene, 0.001 mol % (relative to benzene per single Rh atom) [(η^2^-C_2_H_4_)_2_Rh­(μ-OPiv)]_2_, 240 equiv (relative to Rh) benzoquinone, 960 equiv HOPiv, 50 psig propylene, 170 °C, 2 h. Each data point represents the average of a minimum of three independent experiments, and the error bars represent the standard deviation from multiple experiments.

In contrast to the *para*-benzoquinone derivatives, the trend observed for *ortho*-benzoquinone derivatives appears to follow quinone substituent donor ability, which we approximate using density functional theory (DFT)-calculated p*K*_a1_ values for the corresponding hydroquinone derivatives as reported by Liang and co-workers.^70^ As shown in Figure 7 (left), linear/branched selectivity increases as calculated *ortho*-hydroquinone p*K*_a_ is increased, suggesting that increasing *ortho*-quinone donor ability results in an increase in linear product formation. In contrast, a minimal change in selectivity is observed as a function of *para*-quinone substituent donor ability (Figure 7, right).

**Figure 7:**
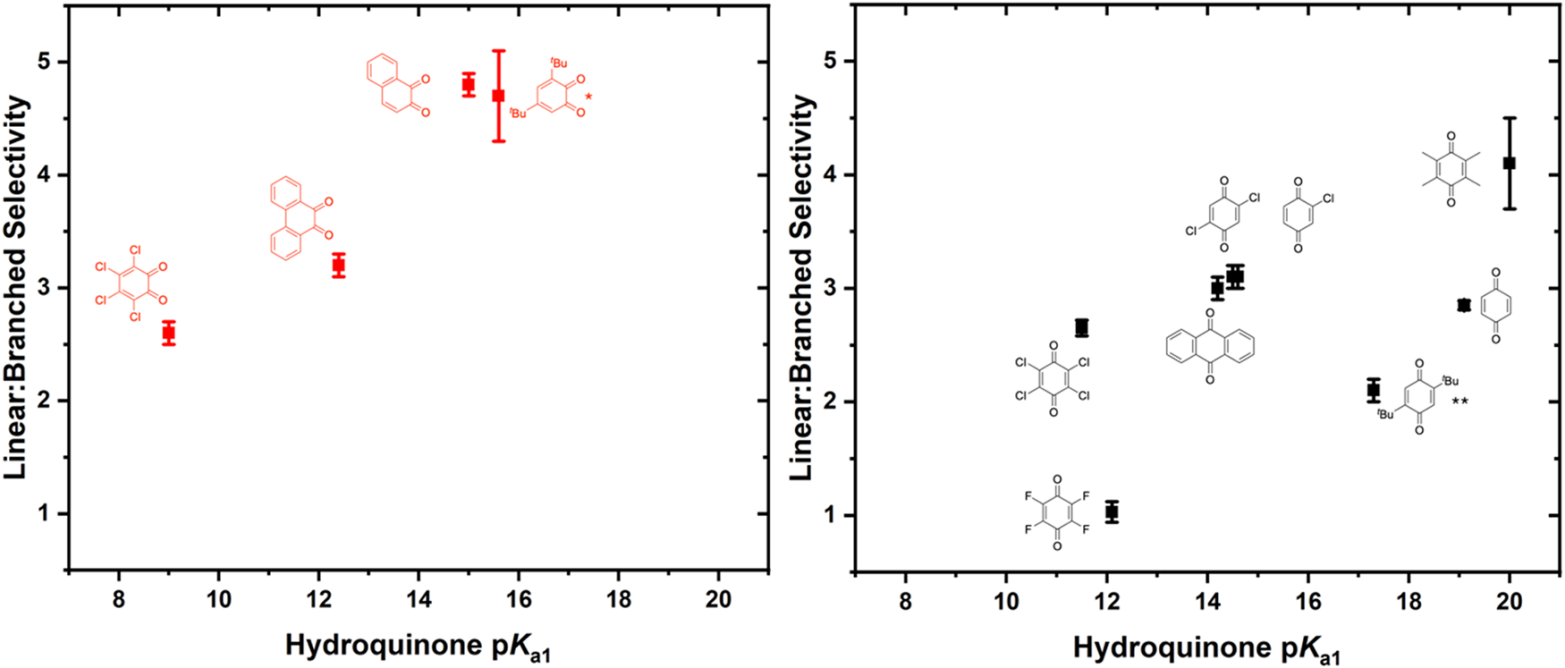
Linear/branched selectivity for benzene propenylation as a function of p*K*_a1_ for the hydroquinone derivatives corresponding to the *ortho-* and *para*-quinones. *p*K*_a1_ of 3,5-dimethyl-*ortho*-benzoquinone was used as an approximation for that of 3,5-di-*tert*-butyl-*ortho*-benzoquinone. **p*K*_a1_ of 2,5-dimethyl-*para*-benzoquinone was used as an approximation for that of 2,5-di-*tert*-butyl-*para*-benzoquinone. Reaction conditions: 7.5 mL of benzene, 0.001 mol % (relative to benzene per single Rh atom) [(η^2^-C_2_H_4_)_2_Rh­(μ-OPiv)]_2_, 240 equiv (relative to Rh) benzoquinone, 960 equiv HOPiv, 50 psig propylene, 170 °C, 2 h. Each data point represents the average of a minimum of three independent experiments, and the error bars represent the standard deviation from multiple experiments.

We speculate that the dependence of linear/branched selectivity on *ortho*-quinone substituent donor ability might be attributable to *ortho*-quinones serving as ligands, and the formed Rh quinone complexes are electronically modulated by the quinone substituent donor ability. This possibility is discussed in more detail below. The lack of a statistically significant trend in linear/branched selectivity for the *para*-benzoquinone derivatives when considering both the *para*-quinone reduction potential and substituent donor ability could suggest that a *para*-quinone ligand is not bound to Rh during steps that determine linear/branched selectivity.

To further probe the influence of quinone identity on selectivity, ortho/meta/para regioselectivity was studied with *tert*-butylbenzene as the arene (Figure 8). Since *tert*-butylbenzene has a sterically encumbered ortho C–H bond, only trace quantities of ortho-alkenylated products are generally observed for transition metal-catalyzed arene alkenylation reactions.^18,19^ The simplification in product distribution due to the lack of *ortho*-*tert*-butylstyrene production can be useful in elucidating the mechanism of arene C–H bond activation: a meta/para selectivity close to 2:1 is generally consistent with a mechanism lacking significant electronic effects, whereas a bias toward the production of para products can indicate that C–H activation possesses electrophilic character.^18,19^ Previously, for monosubstituted arenes, we have demonstrated that the ortho/meta/para selectivity for Rh-catalyzed arene alkenylation is largely insensitive to the donor properties of the substituent, whereas Pd catalysis is more sensitive.^19^ For disubstituted arenes, the Rh catalysis appears more complicated, with experiments and computational results indicating a likely change in mechanism for C–H activation as a function of the substituent identities.^71^ Among *ortho*- and *para*-quinones, no clear trend in meta/para selectivity is observed as a function of the quinone reduction potential (Figure 9). Plotting meta/para selectivity as a function of *E*_1/2_ values for *ortho*-quinones measured in the presence of HOPiv results in a similar trend to *E*_1/2_ measured in the absence of HOPiv (Figure S24).

**Figure 8:**
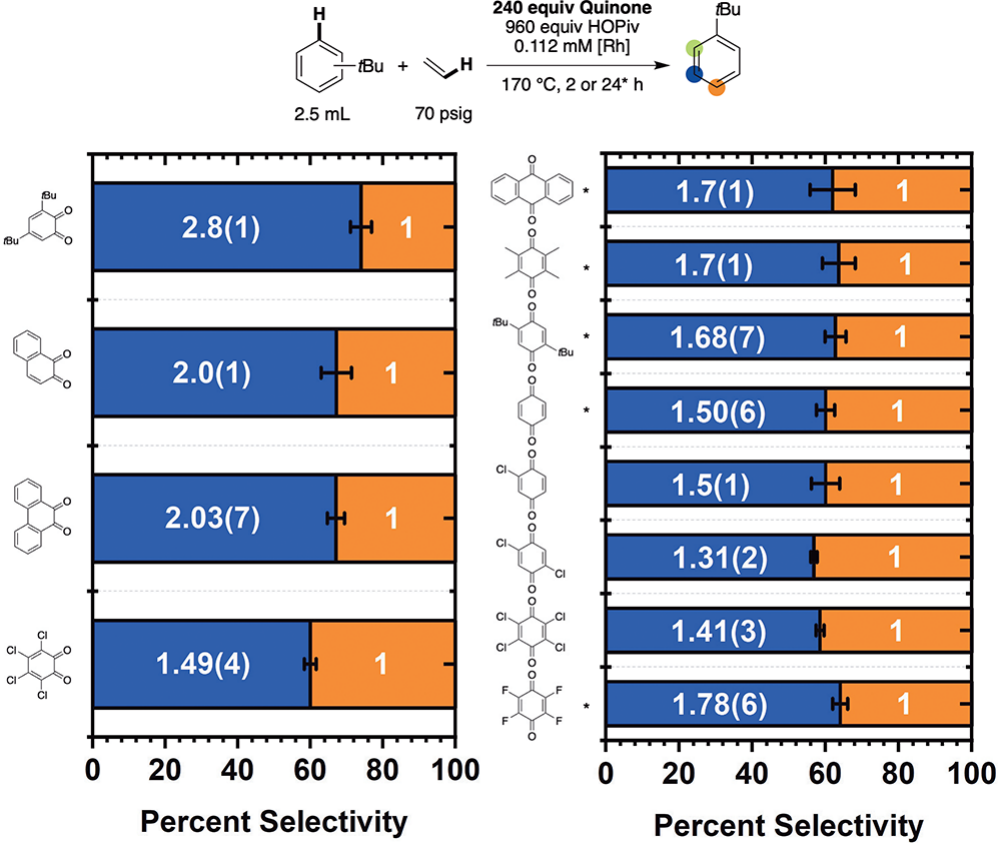
Regioselectivity of *tert*-butylbenzene ethenylation as a function of benzoquinone identity. Reaction conditions: 2.5 mL of t*ert*-butylbenzene, 0.112 mM (relative to a single Rh atom) [(η^2^-C_2_H_4_)_2_Rh­(μ-OPiv)]_2_, 240 equiv (relative to Rh) benzoquinone, 960 equiv of HOPiv, 70 psig ethylene, 170 °C. For quinones marked with asterisks, 24 h reaction times were used; otherwise, 2 h reaction times were used. Each data point represents the average of a minimum of three independent experiments, and the error bars represent the standard deviation from multiple experiments.

**Figure 9:**
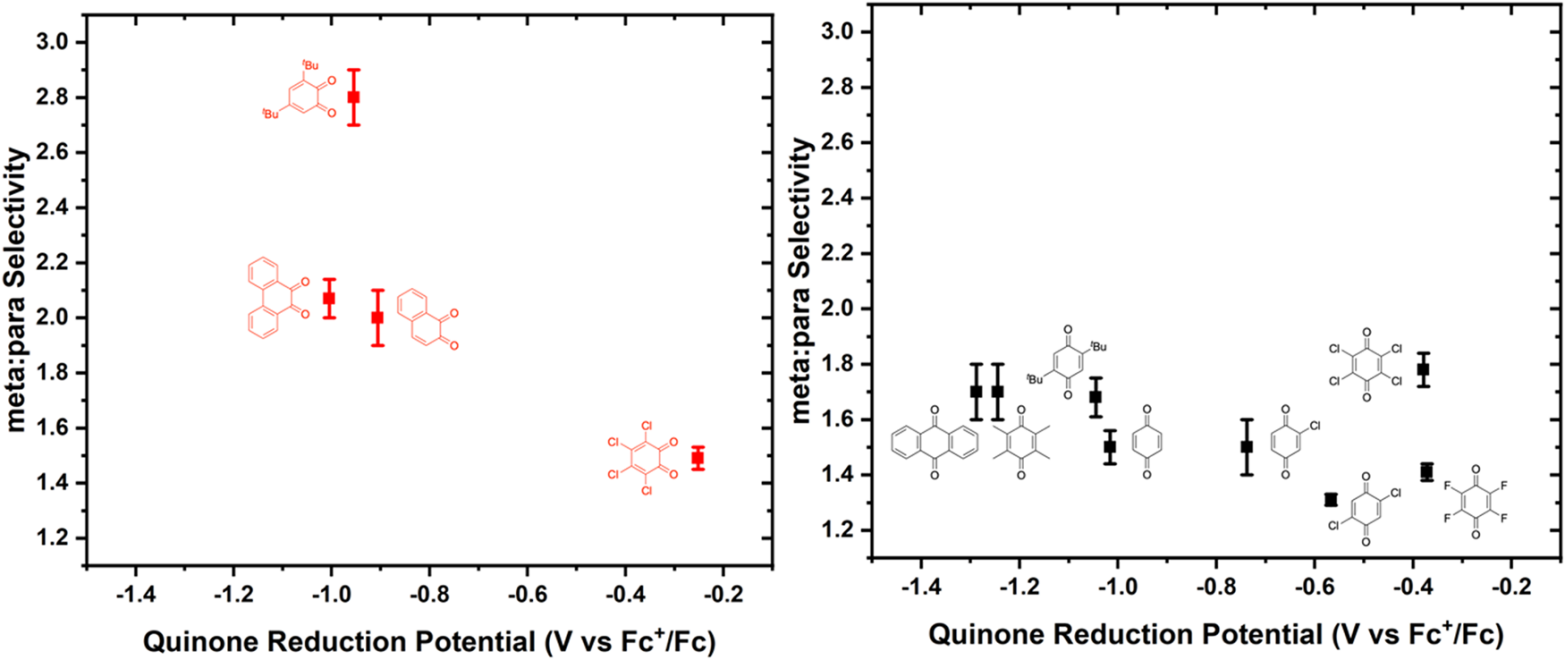
Meta/para selectivity for the ethenylation of *tert*-butylbenzene as a function of the benzoquinone reduction potential. Reaction conditions: 2.5 mL of *tert*-butylbenzene, 0.112 mM (relative to a single Rh atom) [(η^2^-C_2_H_4_)_2_Rh­(μ-OPiv)]_2_, 240 equiv (relative to Rh) benzoquinone, 960 equiv HOPiv, 70 psig ethylene, 170 °C. For quinones marked with asterisks, 24 h reaction times were used; otherwise, 2 h reaction times were used. Each data point represents the average of a minimum of three independent experiments, and the error bars represent the standard deviation from multiple experiments.

Using *ortho*-quinones, 3,5-di-*tert*-butyl-*ortho*-benzoquinone gives a meta/para selectivity of 2.8(1):1, phenanthrene dione and naphthoquinone give selectivity of ∼2:1, and *ortho*-chloranil gives a selectivity of 1.49(4):1. Use of electron-deficient *ortho*-chloranil results in the most significant para selectivity, perhaps indicating that the arene C–H activation step possesses some electrophilic character. When plotting meta/para regioselectivity as a function of hydroquinone p*K*_a1_ corresponding to the *ortho*-benzoquinone derivatives, a general increase in meta selectivity as a function of p*K*_a1_ is observed (Figure 10). With *para*-benzoquinones, no clear trend is observed as a function of substituent donor ability, indicating that *para*-benzoquinones are not bound to Rh during steps that determine meta/para regioselectivity or that bound *para*-benzoquinones do not influence regioselectivity. Previously, the Sanford group reported that *para*-quinones influence ortho/meta/para regioselectivity for Pd-catalyzed anisole arylation and proposed that the quinone η^2^ binds to the Pd center during regioselectivity-determining steps.^72^

**Figure 10:**
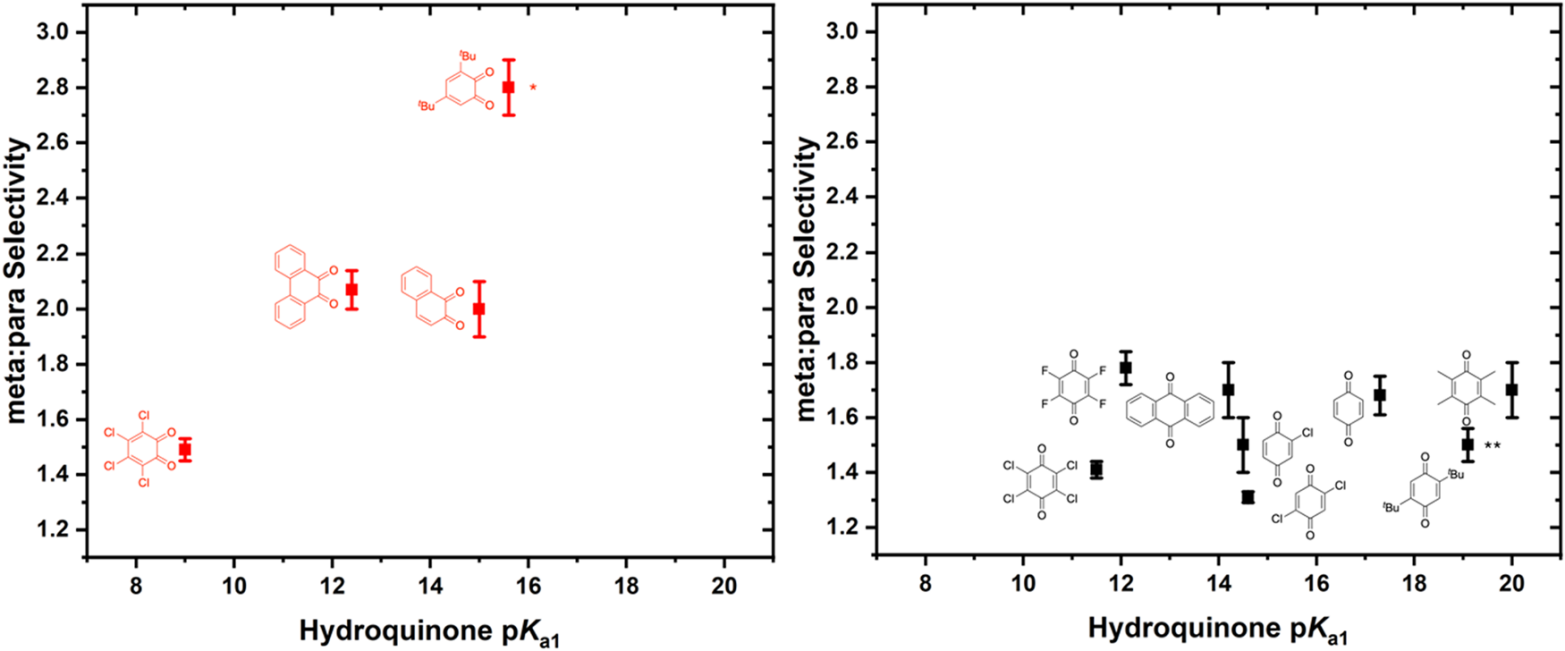
Meta/para selectivity for the ethenylation of *tert*-butylbenzene as a function of p*K*_a1_ of the corresponding hydroquinone derivative. *p*K*_a1_ of 3,5-dimethyl-*ortho*-benzoquinone was used as an approximation for that of 3,5-di-*tert*-butyl-*ortho*-benzoquinone. **p*K*_a1_ of 2,5-dimethyl-*para*-benzoquinone was used as an approximation for that of 2,5-di-*tert*-butyl-*para*-benzoquinone. Reaction conditions: 2.5 mL of *tert*-butylbenzene, 0.112 mM (relative to a single Rh atom) [(η^2^-C_2_H_4_)_2_Rh­(μ-OPiv)]_2_, 240 equiv (relative to Rh) benzoquinone, 960 equiv HOPiv, 70 psig ethylene, 170 °C. For quinones marked with asterisks, 24 h reaction times were used; otherwise, 2 h reaction times were used. Each data point represents the average of a minimum of three independent experiments, and the error bars represent the standard deviation from multiple experiments.

Previously, our group reported that the use of Pd­(OAc)_2_ as a catalyst precursor with Cu­(OPiv)_2_ as the in situ oxidant results in ortho/meta/para regioselectivity that favors alkenylation at positions favored by electrophilic aromatic substitution processes.^19^ For example, the use of toluene as the arene resulted in a 0.5:1:1 ortho/meta/para ratio, while the use of trifluoromethylbenzene resulted in a 0.1:3:1 ortho/meta/para selectivity.^19^ In contrast, Rh catalysis with Cu­(OPiv)_2_ as the oxidant produces minimal ortho product, and a meta/para selectivity ranging from 1:1 to 3:1 with most monosubstituted arenes, a selectivity that is dependent on reaction conditions but that is less sensitive to arene substituent than Pd catalysis with Cu­(OPiv)_2_ as the oxidant.^19,71,73,74^ The observation that variation of *ortho*-quinone substituent electron-withdrawing ability results in a substantial shift toward the production of para products is consistent with *ortho*-quinones serving as a bidentate ligand, which modulates electron density on the Rh center and, thus, the mechanism of arene C–H activation.

To further probe whether the *ortho*-quinone substituent donor ability modulates the mechanism by which Rh activates arene C–H bonds, an intermolecular competition experiment using equimolar quantities of toluene and α,α,α-trifluoro-toluene was performed. Previously, we have used this experiment to differentiate C–H activation reactions with electrophilic character from those that proceed by concerted metalation–deprotonation (CMD) or oxidative addition mechanisms.^18,19^ While electrophilic processes proceed more rapidly with electron-rich arenes (e.g., toluene), processes that operate through concerted metalation–deprotonation can proceed more rapidly with substrates bearing more acidic C–H bonds (e.g., α,α,α-trifluorotoluene).^75–77^ We studied this competition reaction using 3,5-di-*tert*-butyl-*ortho*-benzoquinone, phenanthrene dione, *ortho*-chloranil, and *para*-chloranil as the oxidant, comparing the TOs of functionalized styrene produced after 2 h (Figure 11).

**Figure 11:**
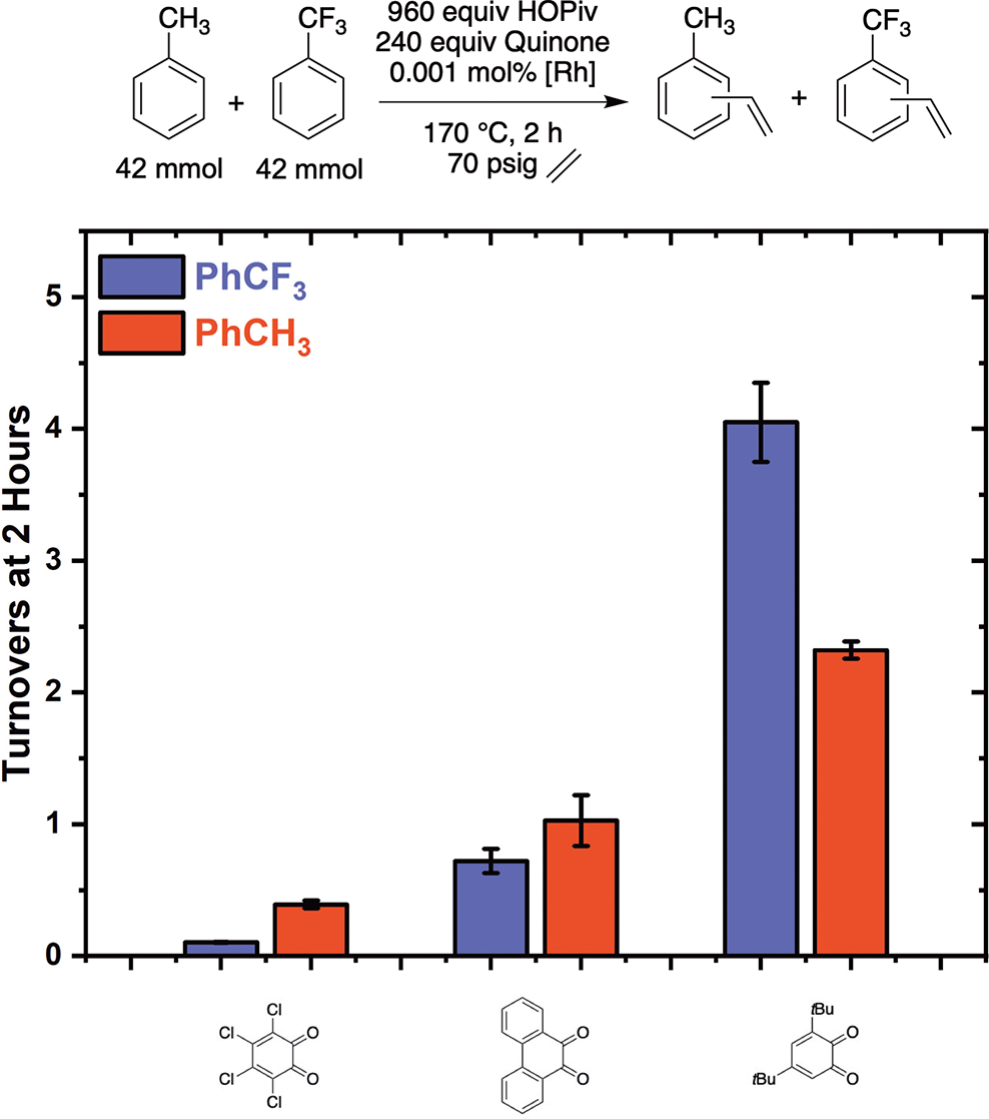
Kinetic competition experiment using equimolar quantities of PhCH_3_ and PhCF_3_ with 3,5-di-*tert*-butyl-*ortho*-benzoquinone, phenanthrene dione, *ortho*-chloranil, or *para*-chloranil as the oxidant. Reaction conditions: 42 mmol PhCH_3_, 42 mmol PhCF_3_, 0.001 mol % (relative to total moles of arene on the basis of single Rh atoms) [(η^2^-C_2_H_4_)_2_Rh­(μ-OPiv)]_2_, 240 equiv (relative to Rh) benzoquinone, 960 equiv HOPiv, 70 psig ethylene, 170 °C. Each data point represents the average of a minimum of three independent experiments, and the error bars represent the standard deviation from multiple experiments.

As shown in Figure 11, with 3,5-di-*tert*-butyl-*ortho*-benzoquinone as the oxidant, α,α,α-trifluorotoluene reacts ∼2-fold more rapidly than toluene. With phenanthrene quinone as the oxidant, toluene and α,α,α-trifluorotoluene react at statistically identical rates, and with *ortho*-chloranil as the oxidant, toluene reacts ∼4-fold more rapidly than α,α,α-trifluorotoluene. Use of *para*-chloranil as the oxidant results in toluene reacting 3.6-fold more rapidly than α,α,α-trifluoro-toluene.

The variation in activity toward toluene versus α,α,α-trifluorotoluene as *ortho*-quinone identity is varied is consistent with the formation of semiquinone or catecholate-ligated Rh complexes under the reaction conditions. The use of 3,5-di-*tert*-butyl-*ortho*-benzoquinone as the oxidant results in a preference for the C–H activation of α,α,α-trifluorotoluene, which bears more acidic C–H bonds than toluene. We speculate that the preference for more acidic bonds originates from the formation of an electron-rich Rh active species that likely activates C–H bonds through a CMD mechanism (Scheme 3).^75,76,78^ Also, since the transition state for CMD contains significant arene deprotonation character, the presence of electron-withdrawing arene substituents can stabilize the partial negative charge.^78,79^ In contrast, the use of *ortho*-chloranil as the oxidant could form a comparatively electron-deficient Rh complex, which might operate through a mechanism bearing a more electrophilic character.^78–80^ The transition state for electrophilic CMD involves a partial positive charge on the arene, which is stabilized by electron-donating arene substituents. The quinone 9,10-phenanthrene dione has an intermediate electron donor ability compared to 3,5-di-*tert*-butyl-*ortho*-benzoquinone and *ortho*-chloranil based on the calculated p*K*_a1_ of its corresponding hydroquinone.^70^ Consistent with its predicted electron donor ability based on calculated p*K*_a1_, the use of 9,10-phenanthrene dione as the oxidant results in statistically identical rates of reaction between α,α,α-trifluorotoluene and toluene. This suggests an intermediate mechanism between the electrophilic CMD observed with *ortho*-chloranil and the proposed CMD mechanism that is observed for 3,5-di-*tert*-butyl-*ortho*-benzoquinone.

**Scheme 3:**
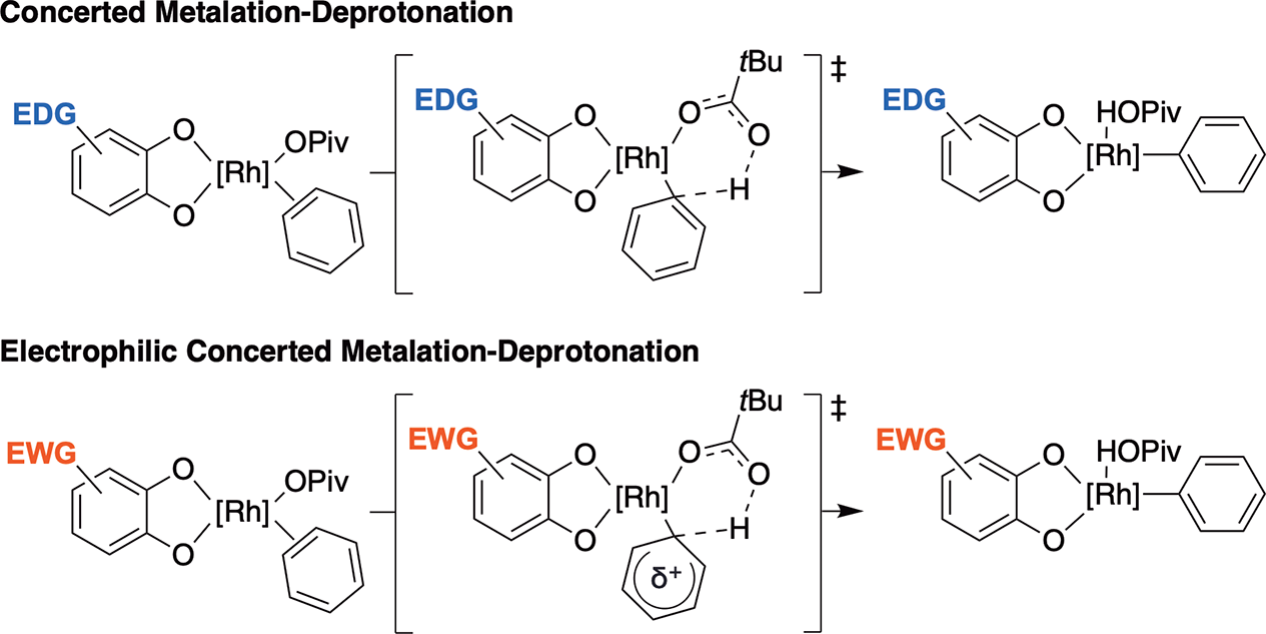
Proposed Mechanistic Differences of Arene C–H Activation between Systems in Which Electron-Rich and Electron-Deficient Benzoquinones Are Used as Oxidants

## Summary and Conclusions

Our results indicate that the use of electron-deficient *ortho*-benzoquinones results in a C–H activation mechanism with more electrophilic character compared to *ortho*-benzoquinones that are relatively more electron-rich, a metric that we approximate using calculated p*K*_a_ values for the corresponding hydroquinones.^70^ The observed trends in the C–H activation mechanism do not seem to follow quinone reduction potential, but rather the donor ability of *ortho*-quinone substituents might play a role. These findings suggest that *ortho*-benzoquinones likely react with [(η^2^-C_2_H_4_)_2_Rh­(μ-OPiv)]_2_ to form Rh­(II) semiquinone or Rh­(III) catecholate complexes (Scheme 4), for which there is literature precedent, and these complexes serve as the active catalysts for arene alkenylation.^81–84^

**Scheme 4:**
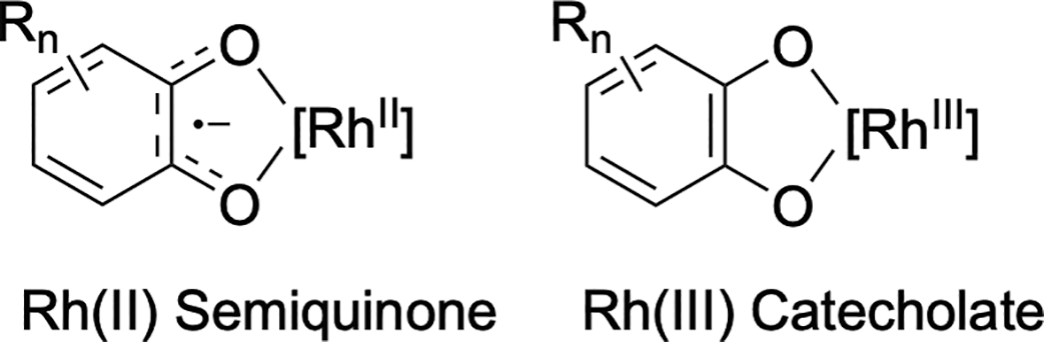
Possible Structures of Complexes Formed upon the Reaction of [(η^2^-C_2_H_4_)_2_Rh­(μ-OPiv)]_2_ with *ortho*-Quinone Derivatives

Rh complexes formed from the reaction of [(η^2^-C_2_H_4_)_2_Rh­(μ-OPiv)]_2_ with 3,5-di-*tert*-butyl-*ortho*-benzoquinone are expected to be more electron-rich than those bearing *ortho*-chloranil as a ligand. The proposed Rh semiquinone or catecholate complexes bear resemblance to Ir­(III) catalysts for arene alkylation reported by the Periana and Goddard groups, which utilized acetylacetonate or tropolonate ligands.^25–27^ The Periana and Goddard groups also reported aerobic Rh-catalyzed styrene production in the presence of acetylacetone, which could form Rh­(acac) complexes.^46^

When probing linear/branched selectivity with propylene as the olefin, increasing the electron-withdrawing ability of quinone substituents results in a decrease in the production of linear products. The observation of higher linear selectivity with more electron-rich proposed Rh complexes mirrors previous findings by our group on Pt-catalyzed arene alkylation. Using cationic bipyridyl Pt­(II) complexes as the catalyst, linear/branched selectivity increased as the 4,4′-bipyridine substituent donor ability was increased.^36^

In contrast to the *ortho*-quinones, for which substantial ortho/meta/para and linear/branched selectivity changes were observed as a function of quinone identity, modulation of *para*-quinone reduction potential and, hence, ligand donor ability produces small changes in ortho/meta/para and linear/branched selectivity. *para*-Benzoquinones typically bind to late transition metals through η^2^ or η^4^ bonding.^85–87^ Previous studies of catalytic processes using *para*-benzoquinone additives found that the addition of quinone substituents can significantly inhibit quinone coordination to an active catalyst, promoting activity.^63^ The Sanford group reported a Pd-catalyzed benzo­(*h*)­quinoline C–H arylation reaction for which the ortho/meta/para regioselectivity with anisole was dependent upon benzoquinone identity.^51,72^ It was proposed that the benzoquinone is coordinated to the Pd center during the regioselectivity-determining steps. Herein, we observe modest changes in linear/branched and ortho/meta/para regioselectivity as the *para*-quinone identity is varied. While additional studies are necessary to determine if *para*-quinones coordinate to Rh under the reaction conditions, the results in this study are consistent with any possible coordination having a negligible effect on regioselectivity patterns.

Taken together, our studies suggest that *ortho*-quinone derivatives likely bind to the active Rh species, resulting in ligand effects. The apparent ligand effects manifest in significant variation in linear/branched and ortho/meta/para regioselectivity with monosubstituted olefins and arenes.

## Experimental Section

### General Considerations

Unless otherwise noted, all synthetic procedures were performed under anaerobic conditions in a dinitrogen-filled glovebox. Glovebox purity was maintained by periodic dinitrogen purges and was monitored by an oxygen analyzer (O_2_ < 15 ppm for all reactions). Pentanes and benzene were obtained from a commercial source and purified using a solvent purification system with activated alumina. Ag­(OPiv)^88^ and [(η^2^-C_2_H_4_)_2_Rh­(μ-Cl)]_2_ ^89^ were synthesized by previously reported procedures. All other reagents were obtained from commercial sources and used as received. High-pressure reactions were performed in custom stainless steel reactors fitted with pressure-relief valves. While heating and pressurized, they were kept behind a blast shield to protect the operator in the event that a pressure-relief valve opened. GC-MS analysis was performed using a Shimadzu GC-MS-QP-2030 Plus system with a 30 m × 20.25 mm RTX-Rxi-5 ms column with a 0.25 μM film thickness. A plot of peak area versus molar ratio gave a regression line using hexamethylbenzene as an internal standard. The slope and correlation coefficient of the regression lines were 0.394 and 0.999 (styrene), 0.238 and 0.993 (vinyl pivalate), 0.232 and 0.999 (benzaldehyde), 0.740 and 0.999 (phenyl pivalate), 0.964 and 0.999 (biphenyl), 0.679 and 0.999 (*trans*-stilbene), 0.562 and 0.998 (1,5-di-*tert*-butylbicyclo­[2.2.2]­oct-5-ene-2,3-dione), 0.348 and 0.999 (3-trifluoromethylstyrene), and 0.357 and 0.999 (3-methylstyrene). For benzene propenylation and *tert*-butylbenzene ethenylation reactions, propenyl benzenes and *tert*-butylstyrenes were identified by their mass spectra, and regioselectivity was quantified by relative peak area.

### Synthesis of [(η^2^-C_2_H_4_)_2_Rh­(μ-OPiv)]_2_

[(η^2^-C_2_H_4_)_2_Rh­(μ-Cl)]_2_ (447 mg, 1.15 mmol, 1 equiv) and Ag­(OPiv) (528 mg, 2.50 mmol, 2.2 equiv) were combined in 50 mL of pentane and stirred in darkness for 4 h to produce a maroon solution. The reaction mixture was filtered through Celite to remove AgCl and excess AgOPiv. Pentane was removed from the filtrate under reduced pressure, and the sticky, dark red product was collected (527 mg, 88% yield). ^1^H NMR (600 MHz, C_6_D_6_): δ 2.90 ppm (br s, 16H), 1.04 ppm (s, 18H); ^13^C NMR (200 mHz, C_6_D_6_): δ 191.19, 50.43, 40.07, 28.09. Anal. Calcd for Rh_2_O_4_C_18_H_34_: C, 41.55; H, 6.58. Found: C, 41.70(3); H, 6.6(1).

### Synthesis of 1,5-di-*tert*-Butylbicyclo­[2.2.2]­oct-5-ene-2,3-dione

Under an atmosphere of dry dinitrogen, four 10 mL vials with stir bars were charged with benzene (10 mL, 112 mmol) and 3,5-di-*tert*-butyl-*ortho*-benzoquinone (200 mg, 0.908 mmol). The vials were used as inserts in four custom-built stainless steel high-pressure reactors equipped with pressure-relief valves. The reactors were sealed, pressurized with ethylene (150 psig), and heated at 170 °C in aluminum blocks on two hot plates. After 24 h, the reactors were cooled to room temperature, the ethylene pressure was slowly released, and the components of the four reactors were combined. Benzene was removed from the combined solution in vacuo, and the resulting yellow-brown oil was purified by column chromatography on a silica gel column using 4:1 hexane/ethyl acetate as the eluent. A dark brown unidentified side product eluted prior to 1,5-di-*tert*-butylbicyclo­[2.2.2]­oct-5-ene-2,3-dione. The solvent mixture was removed in vacuo, yielding a yellow solid, which was washed with cold pentane. X-ray quality crystals were obtained upon storing a saturated pentane solution in a freezer overnight at −34 °C. ^1^H NMR (800 MHz, CD_2_Cl_2_): δ 5.98 ppm (s, 1H), 3.52 (q, 1H, Hz), 2.05 (m, 1H), 1.97 (td, *J*_HH_ = 11.7, 10.1, 5.4 Hz, 1H), 1.81 (td, *J*_HH_ = 12.9, 4.4 Hz, 1H), 1.71 (tdd, *J*_HH_ = 12.7, 5.5, 3.0 Hz, 1H), 1.11 ppm (br, 9H), 1.09 (s, 9H); ^13^C NMR (201 mHz, CD_2_Cl_2_): δ 191.9, 191.4, 153.8, 123.0, 58.0, 48.91, 36.1, 33.1, 27.8, 25.9, 23.5, 23.4. Yield: 104 mg, 11%.

### Kinetics of Benzene Ethenylation

Under an atmosphere of dry dinitrogen, three 10 mL vials with stir bars were charged with benzene (7.5 mL, 84.6 mmol), [(η^2^-C_2_H_4_)_2_Rh­(μ-OPiv)]_2_ (0.21 mg, 0.846 μmol, 0.001 mol % relative to benzene on the basis of single Rh atoms), HOPiv (82.9 mg, 0.812 mmol, 960 equiv relative to single Rh atom), and benzoquinone derivative (0.202 mmol, 240 equiv relative to single Rh atom). The filled vials were used as inserts in custom-built stainless steel reactors equipped with pressure-relief valves. The reactors were sealed, pressurized with 70 psig of ethylene, and heated at 170 °C in an aluminum block on a hot plate. At each time interval, the reactors were removed from the hot plate and cooled to room temperature by placing the hot reactors in a room-temperature aluminum block. Upon cooling, the reactors were opened and sampled using a long needle under a flow of dinitrogen. Next, 100 μL aliquots of the reaction solutions were measured with a microsyringe, diluted in 0.5 mL of ethyl acetate, and combined with 50 μL of an 11.1 mM solution of hexamethylbenzene to give 100 equiv of external standard hexamethylbenzene relative to a single Rh atom. The benzene solutions were washed with a saturated aqueous solution of NaOH, and the organic phases were analyzed by GC-MS.

### Selectivity of Benzene Propenylation

Under an atmosphere of dry dinitrogen, three 10 mL vials with stir bars were charged with benzene (7.5 mL, 84.6 mmol), [(η^2^-C_2_H_4_)_2_Rh­(μ-OPiv)]_2_ (0.21 mg, 0.846 μmol, 0.001 mol % relative to benzene on the basis of single Rh atoms), HOPiv (82.9 mg, 0.812 mmol, 960 equiv relative to single Rh atom), and benzoquinone derivative (0.202 mmol, 240 equiv relative to single Rh atom). The filled vials were used as inserts in custom-built stainless steel reactors equipped with pressure-relief valves. The reactors were sealed, pressurized with 50 psig of propylene, and heated at 170 °C in an aluminum block on a hot plate. After 2 h, the reactors were removed from the hot plate and cooled to room temperature by placing the hot reactors in a room-temperature aluminum block. Upon cooling, the reactors were opened and sampled using a long needle under a flow of dinitrogen. Next, 100 μL aliquots of the reaction solutions were measured with a microsyringe, diluted in 0.5 mL of ethyl acetate, and combined with 50 μL of an 11.1 mM solution of hexamethylbenzene to give 100 equiv of external standard hexamethylbenzene relative to single Rh atoms. The benzene solutions were washed with a saturated aqueous solution of NaOH, and the organic phases were analyzed by GC-MS.

### Selectivity of *tert*-Butylbenzene Ethenylation

Under an atmosphere of dry dinitrogen, three 10 mL vials with stir bars were charged with *tert*-butylbenzene (2.5 mL, 16.15 mmol), [(η^2^-C_2_H_4_)_2_Rh­(μ-OPiv)]_2_ (0.7 mg, 0.282 μmol, 0.112 mM on the basis of single Rh atoms), HOPiv (27.6 mg, 0.27 mmol, 960 equiv relative to a single Rh atom), and a benzoquinone derivative (0.067 mmol, 240 equiv relative to a single Rh atom). The filled vials were used as inserts in custom-built stainless steel reactors equipped with pressure-relief valves. The reactors were sealed, pressurized with 70 psig of ethylene, and heated at 170 °C in an aluminum block on a hot plate. After 2 h, the reactors were removed from the hot plate and cooled to room temperature by placing the hot reactors in a room-temperature aluminum block. Upon cooling, the reactors were opened and sampled using a long needle under a flow of dinitrogen. Next, 100 μL aliquots of the reaction solutions were measured with a microsyringe, diluted in 0.5 mL of ethyl acetate, and combined with 50 μL of an 11.1 mM solution of hexamethylbenzene to give 100 equiv of external standard hexamethylbenzene relative to a single Rh atom. The benzene solutions were washed with a saturated aqueous solution of NaOH, and the organic phases were analyzed by GC-MS.

## Supplementary Material



## Notes

Additional experimental details, including electrochemical data and representative GC-MS chromatograms (PDF)
