# Supplementary material for: Rhodium-Catalyzed Arene Alkenylation Using Benzoquinone Derivatives as Oxidants
Source: Organometallics. 2026 Feb 10;45(4):504–15. doi: 10.1021/acs.organomet.5c00500 (PMC12933888; doi:10.1021/acs.organomet.5c00500)
Supplement: Supplementary file 1 [file om5c00500_si_001.pdf]

Supporting Information

Rhodium Catalyzed Arene Alkenylation using Benzoquinone Derivatives as the Oxidant

Marc T. Bennett,[†] Marina Goupalova,[†] Christopher M. Chapman,[†] Diane A. Dickie,[†] and T. Brent Gunnoe[†]*

[†]Department of Chemistry, University of Virginia, Charlottesville, Virginia 22904

*Correspondence to: tbg7h@virginia.edu

Table of Contents

Representative GC-MS chromatogram for benzene ethenylation catalyzed by $[(\eta^2\text{-}C_2H_4)_2Rh(\mu\text{-}OPiv)]_2$……………………………………………………………………………………S3

GC-MS calibration curves for vinyl pivalate, styrene, biphenyl,*trans*-stilbene and 1,5-di-*tert*-butylbicyclo[2.2.2]oct-5-ene-2,3 dione ……………………………………………………………S3

Representative GC-MS chromatogram for benzene propenylation catalyzed by $[(\eta^2\text{-}C_2H_4)_2Rh(\mu\text{-}OPiv)]_2$……………………………………………………………………………………S4

Representative GC-MS chromatogram for *tert*-butylbenzene ethenylation catalyzed by $[(\eta^2\text{-}C_2H_4)_2Rh(\mu\text{-}OPiv)]_2$……………………………………………………………………………………S4

GC-MS calibration curves for 3-trifluoromethylstyrene and 3-methylstyrene……………………………S4

Reduction potentials for *ortho*-benzoquinone derivatives………………………………………………S5

Reduction potentials for *para*-benzoquinone derivatives…………………………………………………S5

Cyclic voltammogram for 9,10-phenanthrene dione……………………………………………………S6

Cyclic voltammogram for 1,2-napthoquinone……………………………………………………………S6

Cyclic voltammogram for 3,5-di-*tert*-butyl-*ortho*-benzoquinone…………………………………………S7

Cyclic voltammogram for *ortho*-chloranil…………………………………………………………………S7

Cyclic voltammogram for 9,10-anthraquinone……………………………………………………………S8

Cyclic voltammogram for tetramethyl-*para*-benzoquinone………………………………………………S8

Cyclic voltammogram for 2,5-di-*tert*-butyl-*para*-benzoquinone…………………………………………S9

Cyclic voltammogram for *para*-benzoquinone……………………………………………………………S9

Cyclic voltammogram for 2-chloro-*para*-benzoquinone…………………………………………………S10

Cyclic voltammogram for 2,5-di-chloro-*para*-benzoquinone……………………………………………S10

Cyclic voltammogram for *para*-chloranil…………………………………………………………………S11

Cyclic voltammogram for *para*-fluoranil…………………………………………………………………S11

Redox potentials for *ortho*-benzoquinone derivatives in the presence of HOPiv…………………………S12

Cyclic voltammograms of 9,10-phenanthrene dione as a function of HOPiv loading……………………S12

Cyclic voltammograms of naphthoquinone as a function of HOPiv loading………………………………S13

Cyclic voltammograms of 3,5-di-*tert*-butyl-*ortho*-benzoquinone as a function of HOPiv loading…………S14

Cyclic voltammograms of *ortho*-chloranil as a function of HOPiv loading………………………………S15

Benzene ethenylation turnover frequency as a function of benzoquinone redox potential in the presence of HOPiv……………………………………………………………………………………………S16

Linear to branched selectivity for benzene propenylation as a function of benzoquinone redox potential in the presence of HOPiv……………………………………………………………………………………S17

Meta to para selectivity for *tert*-butylbenzene ethenylation as a function of benzoquinone redox potential in the presence of HOPiv……………………………………………………………………………………S18

^{1}H NMR spectrum of $[(\eta^2\text{-}C_2H_4)_2Rh(\mu\text{-}OPiv)]_2$……………………………………………………S19

^{13}C NMR spectrum of $[(\eta^2\text{-}C_2H_4)_2Rh(\mu\text{-}OPiv)]_2$…………………………………………………S20

^{1}H NMR spectrum of 1,5-di-*tert*-butylbicyclo[2.2.2]oct-5-ene-2,3-dione………………………………S21

^{13}C NMR spectrum of 1,5-di-*tert*-butylbicyclo[2.2.2]oct-5-ene-2,3-dione ……………………………S22

Mass spectrum of 1,5-di-*tert*-butylbicyclo[2.2.2]oct-5-ene-2,3-dione……………………………………S23

Mass spectrum of 1,4,5,6-tetrachlorobicyclo[2.2.2]oct-5-ene-2,3-dione…………………………………S24

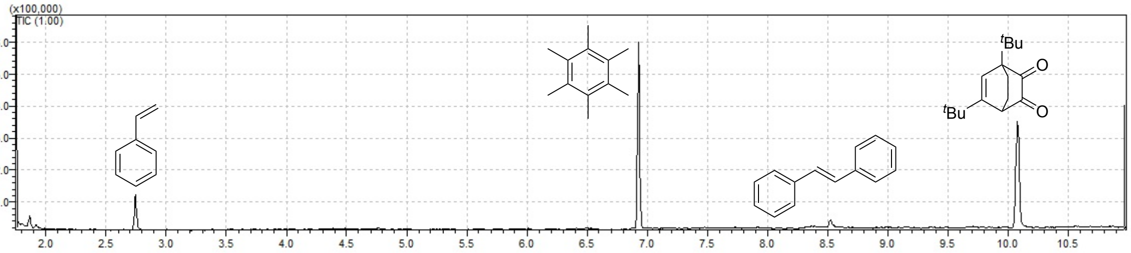

Figure S1. Representative GC-MS chromatogram for benzene ethenylation catalyzed by $[(\eta^2\text{-}C_2H_4)_2Rh(\mu\text{-}OPiv)]_2$ with 3,5-di-*tert*-butyl-*ortho*-benzoquinone as the in situ oxidant.

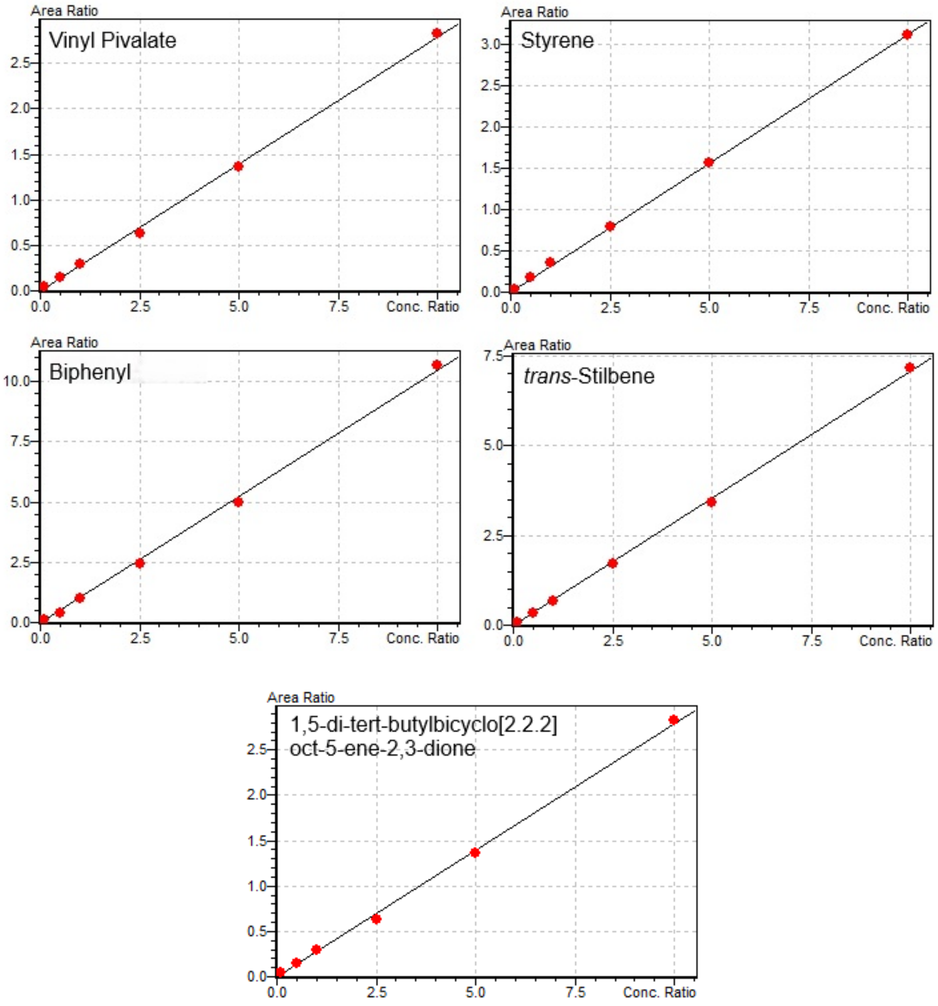

Figure S2. GC-MS calibration curves for vinyl pivalate, styrene, biphenyl,*trans*-stilbene, and 1,5-di-*tert*-butylbicyclo[2.2.2]oct-5-ene-2,3-dione.

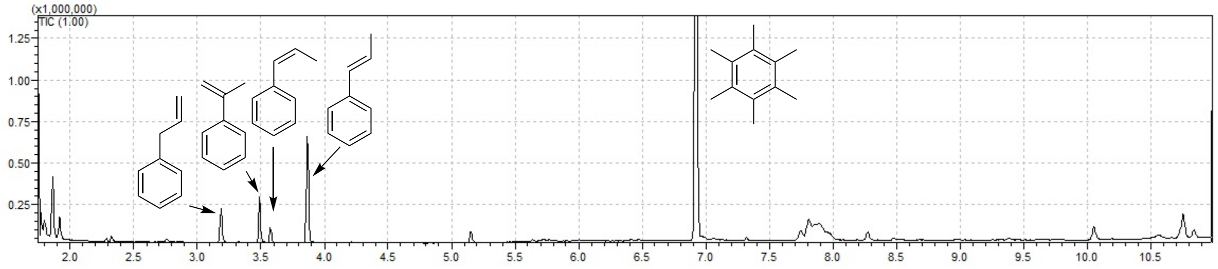

Figure S3. Representative GC-MS chromatogram for benzene propenylation catalyzed by $[(\eta^2\text{-}C_2H_4)_2Rh(\mu\text{-}OPiv)]_2$ with 3,5-di-*tert*-butyl-*ortho*-benzoquinone as the in situ oxidant.

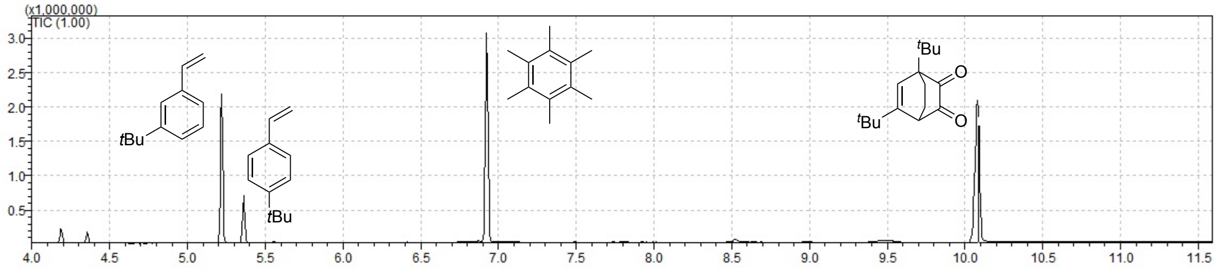

Figure S4. Representative GC-MS chromatogram for *tert*-butylbenzene ethenylation catalyzed by $[(\eta^2\text{-}C_2H_4)_2Rh(\mu\text{-}OPiv)]_2$ with 3,5-di-*tert*-butyl-*ortho*-benzoquinone as the in situ oxidant.

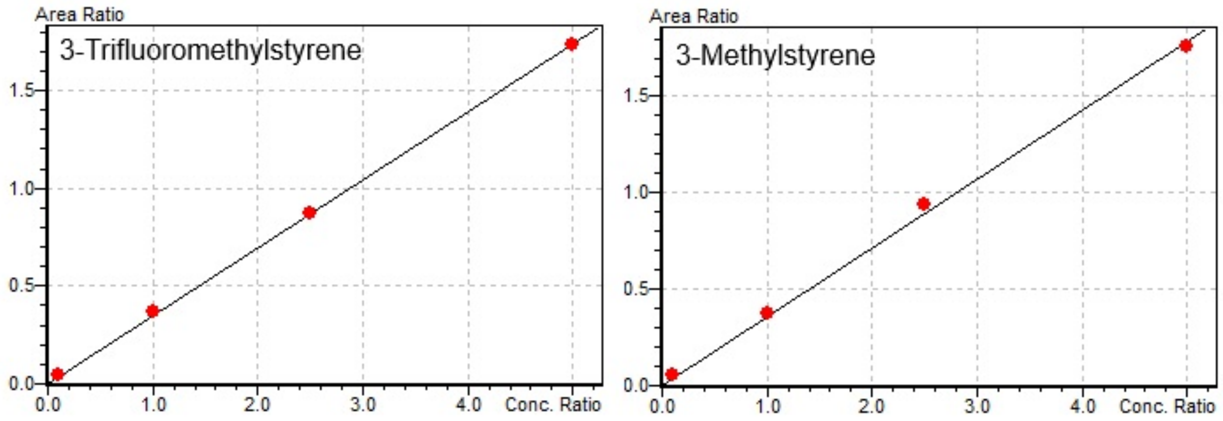

Figure S5. GC-MS calibration curves for 3-trifluoromethylstyrene and 3-methylstyrene.

Table S1. Reduction potentials for *ortho*-benzoquinone derivatives in the absence of HOPiv.

**Benzoquinone**	**$E_{1/2}$ (1) (V vs. Fc^+/Fc)**	**$E_{1/2}$ (2) (V vs. Fc^+/Fc)**
9,10-Phenanthrene Dione	-1.005	-1.656
1,2-Naphthoquinone	-0.906	-1.316
3,5-di-*tert*-butyl-*ortho*-benzoquinone	-0.955	-1.428
ortho-chloranil	-0.252	-1.020

Table S2. Reduction potentials for *para*-benzoquinone derivatives in the absence of HOPiv.

**Benzoquinone**	**$E_{1/2}$ (1) (V vs. Fc^+/Fc)**	**$E_{1/2}$ (2) (V vs. Fc^+/Fc)**
Anthraquinone	-1.288	-1.888
Tetramethyl *para*-benzoquinone	-1.245	-1.899
2,5-di-*tert*-butyl-*para*-benzoquinone	-1.045	-1.676
para-benzoquinone	-1.016	-1.681
2-chloro-*para*-benzoquinone	-0.738	-1.491
2,5-di-chloro-*para*-benzoquinone	-0.567	-1.282
para-fluoranil	-0.379	-1.204
para-chloranil	-0.372	-1.186

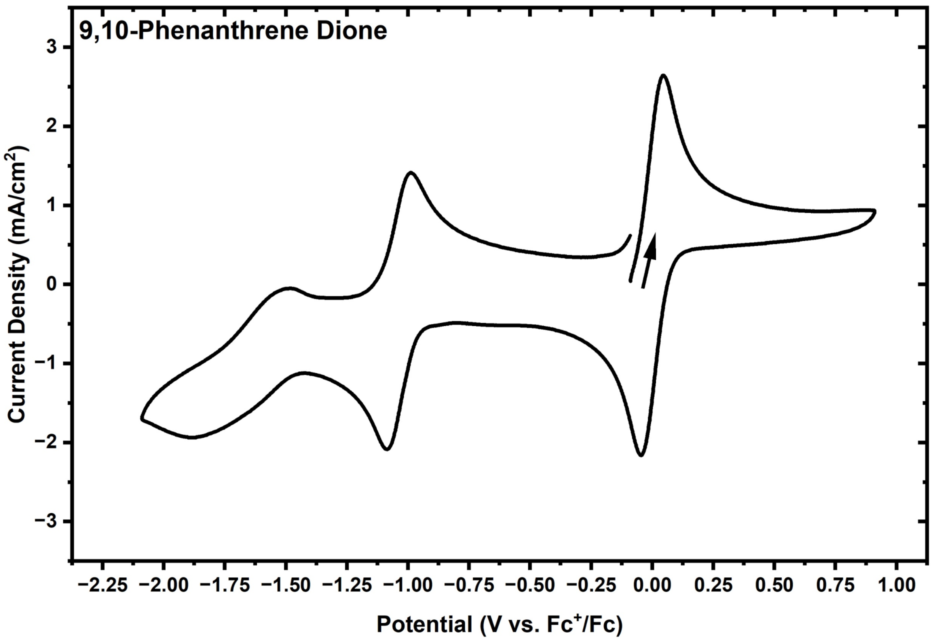

Figure S6. Cyclic voltammogram for 9,10-phenanthrene dione in MeCN solution with 0.1 M [(n-Bu)$_4$N][PF_6] as the supporting electrolyte and ferrocene as an internal reference. Reference electrode: 0.01 M Ag/Ag(NO_3) with 0.1 M [(n-Bu)$_4$N][PF_6] in MeCN with a CoralPor™ frit and Ag wire, working electrode: glassy carbon, counter electrode: Pt wire, scan rate: 0.5 V/s.

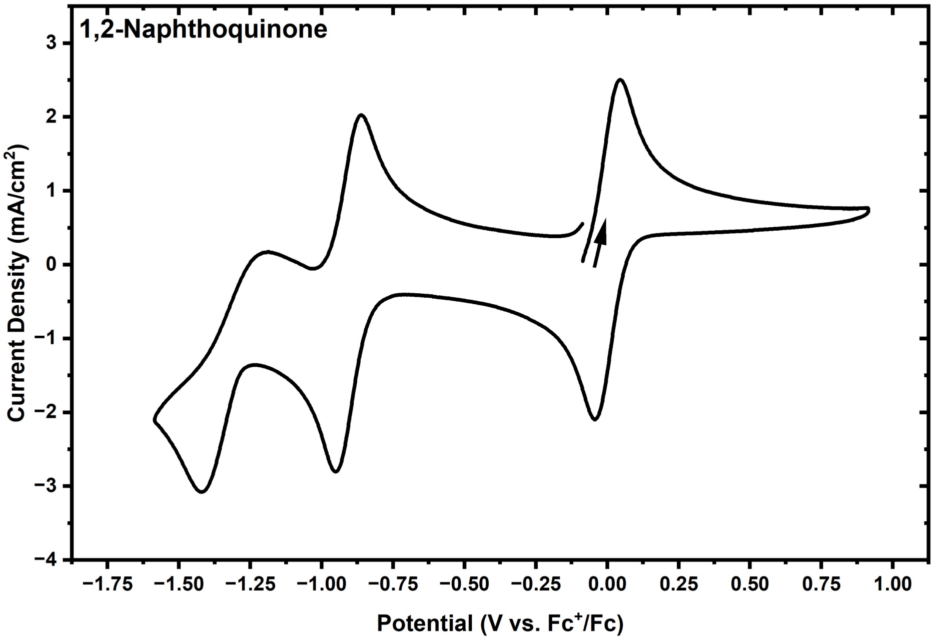

Figure S7. Cyclic voltammogram for 1,2-napthoquinone in MeCN solution with 0.1 M [(n-Bu)$_4$N][PF_6] as the supporting electrolyte and ferrocene as an internal reference. Reference electrode: 0.01 M Ag/Ag(NO_3) with 0.1 M [(n-Bu)$_4$N][PF_6] in MeCN with a CoralPor™ frit and Ag wire, working electrode: glassy carbon, counter electrode: Pt wire, scan rate: 0.5 V/s.

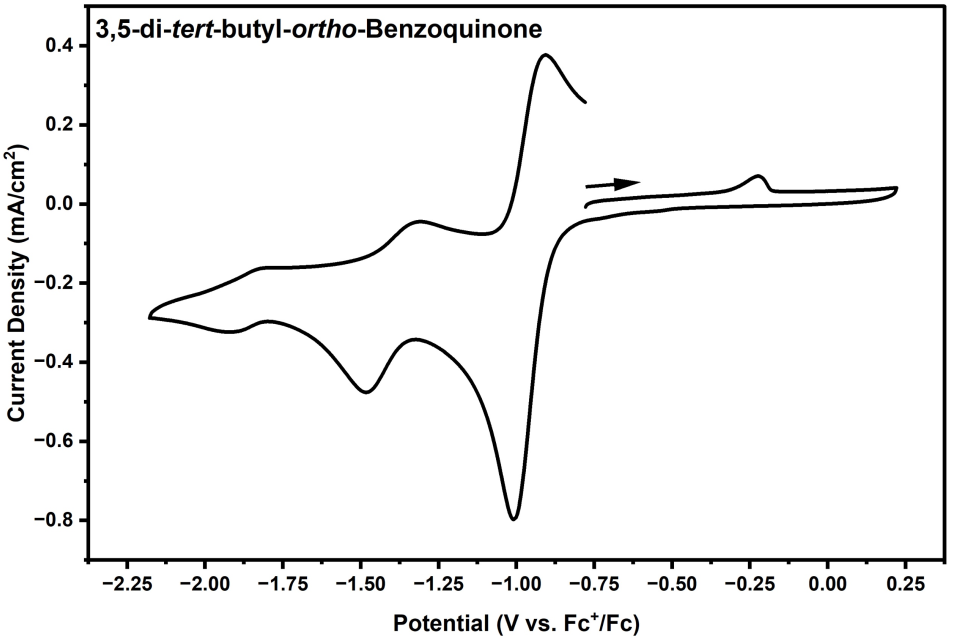

Figure S8. Cyclic voltammogram for 3,5-di-*tert*-butyl-*ortho*-benzoquinone in MeCN solution with 0.1 M [(*n*-Bu)$_4$N][PF_6] as the supporting electrolyte and ferrocene as an internal reference. Reference electrode: 0.01 M Ag/Ag(NO_3) with 0.1 M [(*n*-Bu)$_4$N][PF_6] in MeCN with a CoralPor™ frit and Ag wire, working electrode: glassy carbon, counter electrode: Pt wire, scan rate: 0.1 V/s.

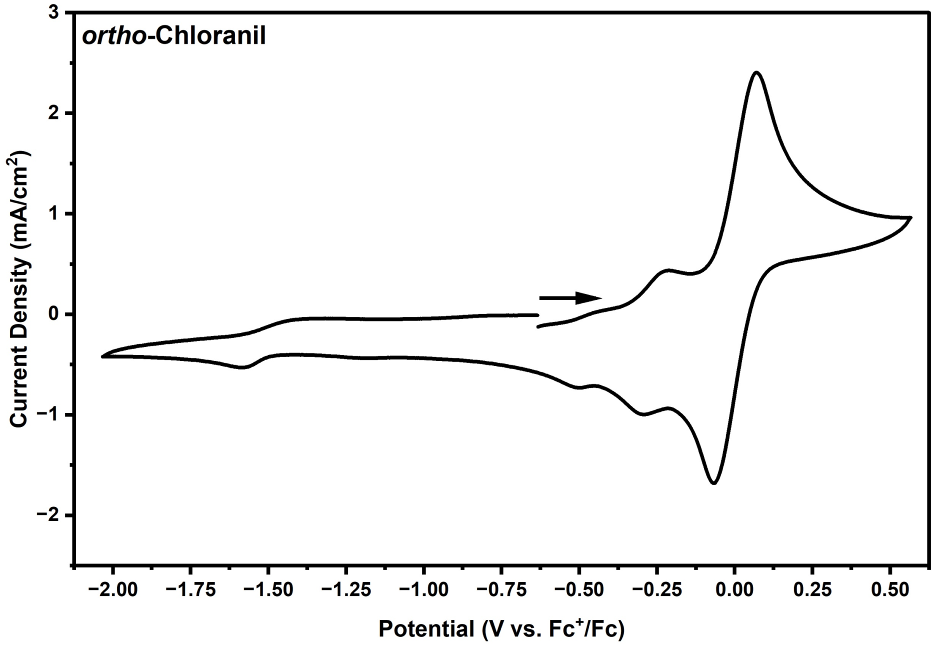

Figure S9. Cyclic voltammogram for *ortho*-chloranil in MeCN solution with 0.1 M [(*n*-Bu)$_4$N][PF_6] as the supporting electrolyte and ferrocene as an internal reference. Reference electrode: 0.01 M Ag/Ag(NO_3) with 0.1 M [(*n*-Bu)$_4$N][PF_6] in MeCN with a CoralPor™ frit and Ag wire, working electrode: glassy carbon, counter electrode: Pt wire, scan rate: 0.2 V/s.

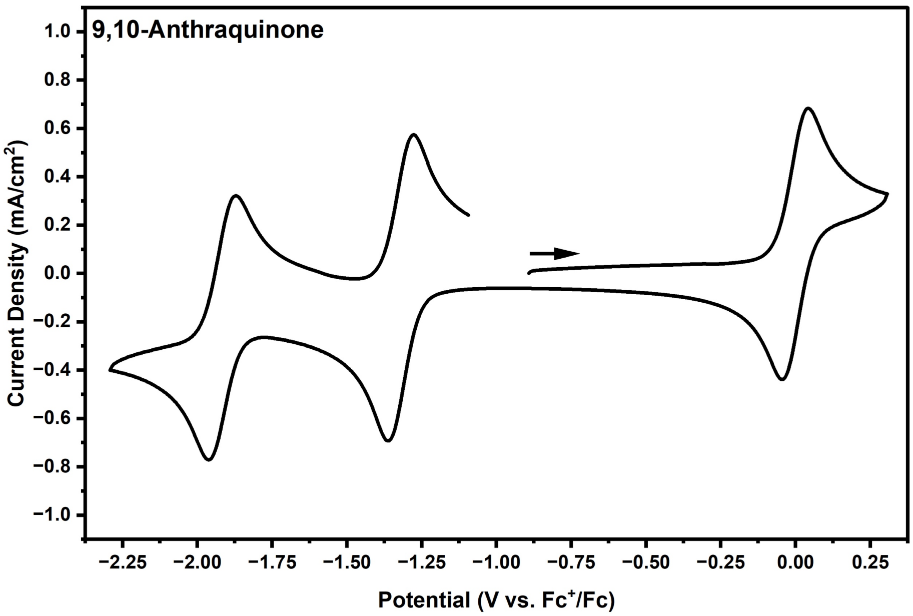

Figure S10. Cyclic voltammogram for 9,10-anthraquinone in MeCN solution with 0.1 M [(*n*-Bu)$_4$N][PF_6] as the supporting electrolyte and ferrocene as an internal reference. Reference electrode: 0.01 M Ag/Ag(NO_3) with 0.1 M [(*n*-Bu)$_4$N][PF_6] in MeCN with a CoralPor™ frit and Ag wire, working electrode: glassy carbon, counter electrode: Pt wire, scan rate: 0.2 V/s.

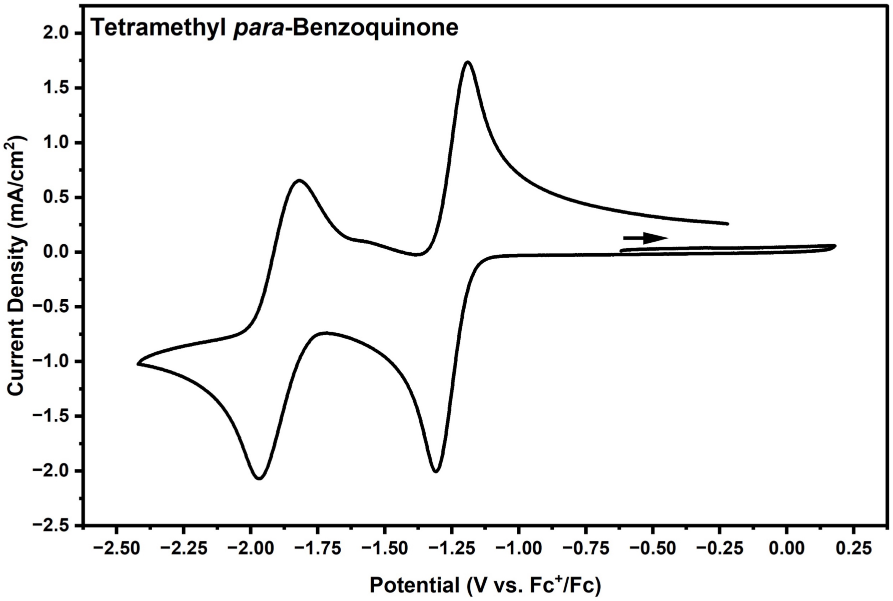

Figure S11. Cyclic voltammogram for tetramethyl-*para*-benzoquinone in MeCN solution with 0.1 M [(*n*-Bu)$_4$N][PF_6] as the supporting electrolyte. Ferrocene was not used as an internal reference in this experiment, and was measured the same day in a separate experiment, and found to have an $E_{1/2}$ at 0.619 V vs Ag/AgNO_3. Reference electrode: 0.01 M Ag/Ag(NO_3) with 0.1 M [(*n*-Bu)$_4$N][PF_6] in MeCN with a CoralPor™ frit and Ag wire, working electrode: glassy carbon, counter electrode: Pt wire, scan rate: 0.5 V/s.

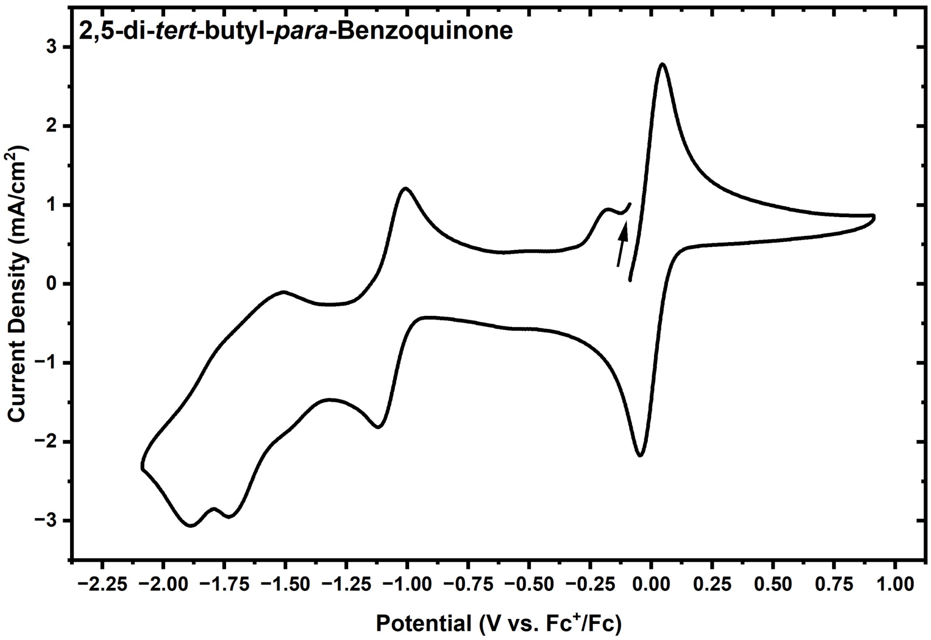

Figure S12. Cyclic voltammogram for 2,5-di-*tert*-butyl-*para*-benzoquinone in MeCN solution with 0.1 M [(n-Bu)$_4$N][PF$_6$] as the supporting electrolyte and ferrocene as an internal reference. Reference electrode: 0.01 M Ag/Ag(NO$_3$) with 0.1 M [(n-Bu)$_4$N][PF$_6$] in MeCN with a CoralPor™ frit and Ag wire, working electrode: glassy carbon, counter electrode: Pt wire, scan rate: 0.5 V/s.

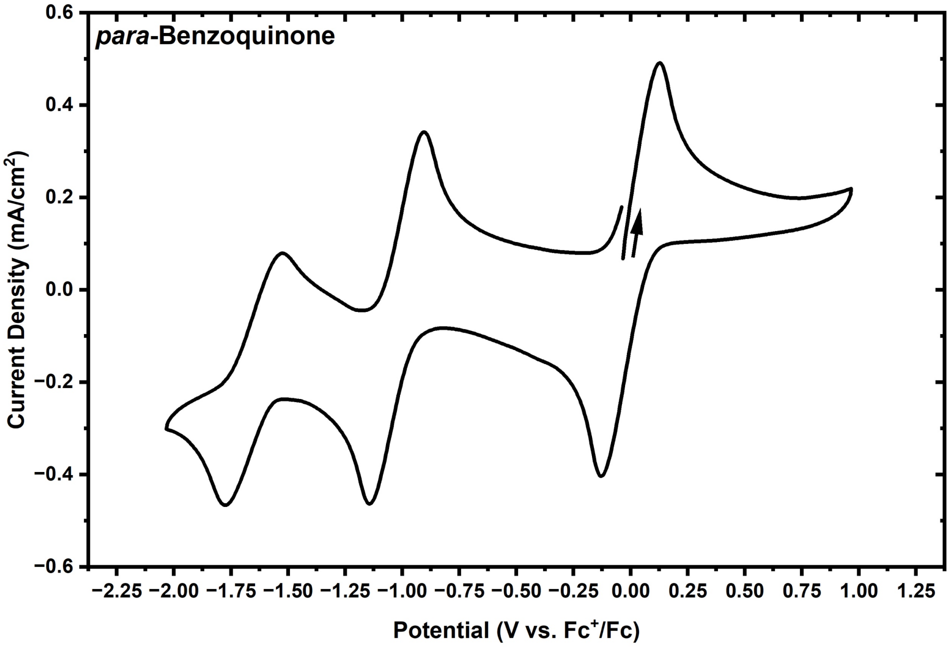

Figure S13. Cyclic voltammogram for *para*-benzoquinone in MeCN solution with 0.1 M [(n-Bu)$_4$N][PF$_6$] as the supporting electrolyte and ferrocene as an internal reference. Reference electrode: 0.01 M Ag/Ag(NO$_3$) with 0.1 M [(n-Bu)$_4$N][PF$_6$] in MeCN with a CoralPor™ frit and Ag wire, working electrode: glassy carbon, counter electrode: Pt wire, scan rate: 0.5 V/s.

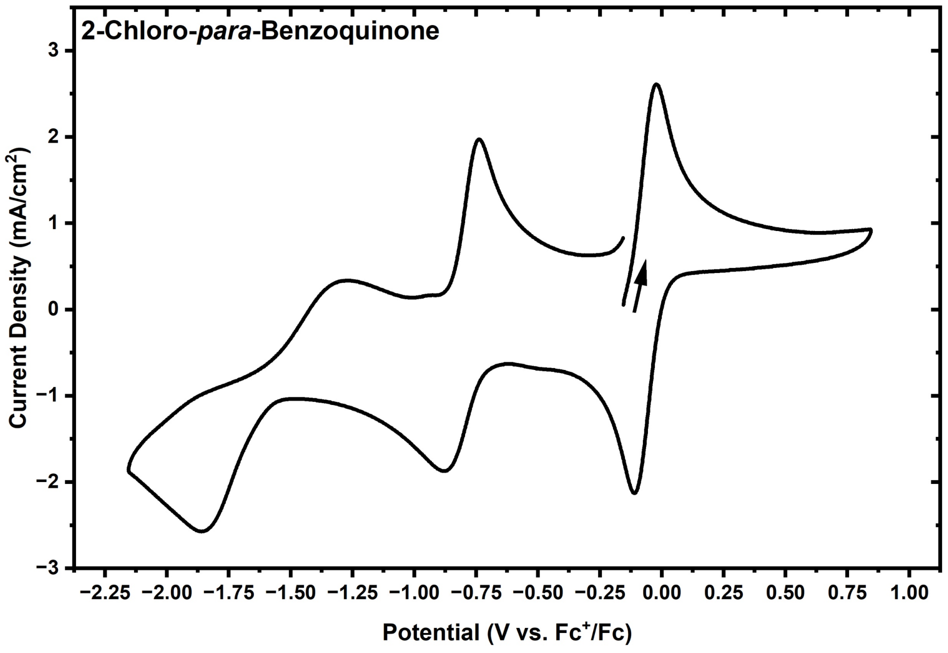

Figure S14. Cyclic voltammogram for 2-chloro-*para*-benzoquinone in MeCN solution with 0.1 M [(n-Bu)$_4$N][PF_6] as the supporting electrolyte and ferrocene as an internal reference. Reference electrode: 0.01 M Ag/Ag(NO_3) with 0.1 M [(n-Bu)$_4$N][PF_6] in MeCN with a CoralPor™ frit and Ag wire, working electrode: glassy carbon, counter electrode: Pt wire, scan rate: 1 V/s.

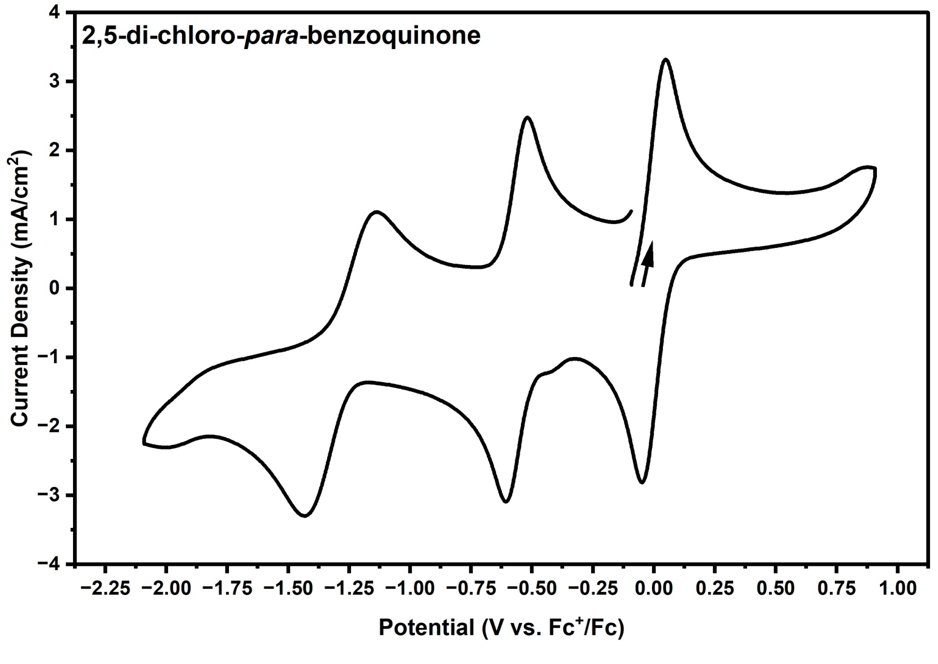

Figure S15. Cyclic voltammogram for 2,5-di-chloro-*para*-benzoquinone in MeCN solution with 0.1 M [(n-Bu)$_4$N][PF_6] as the supporting electrolyte and ferrocene as an internal reference. Reference electrode: 0.01 M Ag/Ag(NO_3) with 0.1 M [(n-Bu)$_4$N][PF_6] in MeCN with a CoralPor™ frit and Ag wire, working electrode: glassy carbon, counter electrode: Pt wire, scan rate: 1 V/s.

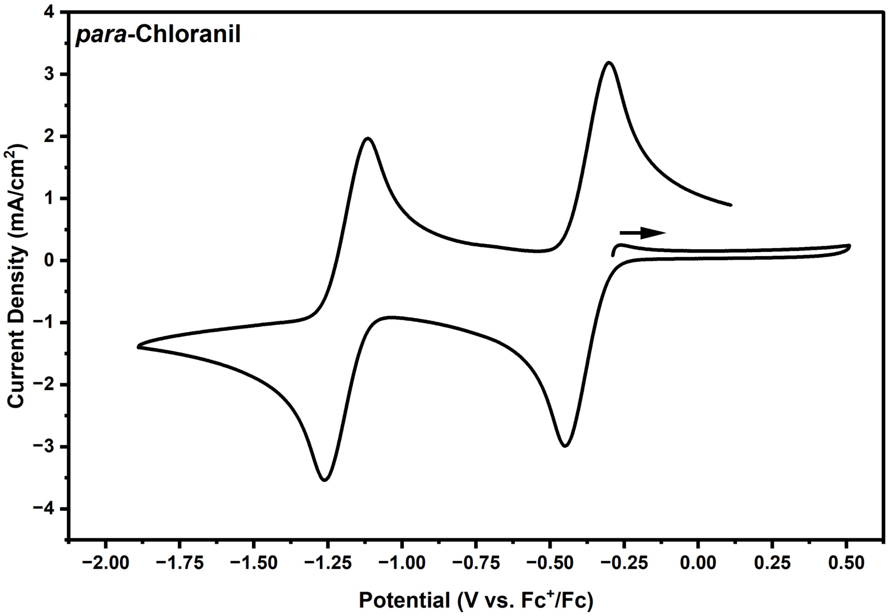

Figure S16. Cyclic voltammogram for *para*-chloranil in MeCN solution with 0.1 M [(*n*-Bu)$_4$N][PF$_6$] as the supporting electrolyte. Ferrocene was not used as an internal reference in this experiment and was measured the same day in a separate experiment and found to have an $E_{1/2}$ at 0.291 V vs Ag/AgNO$_3$. Reference electrode: 0.01 M Ag/Ag(NO$_3$) with 0.1 M [(*n*-Bu)$_4$N][PF$_6$] in MeCN with a CoralPor™ frit and Ag wire, working electrode: glassy carbon, counter electrode: Pt wire, scan rate: 0.5 V/s.

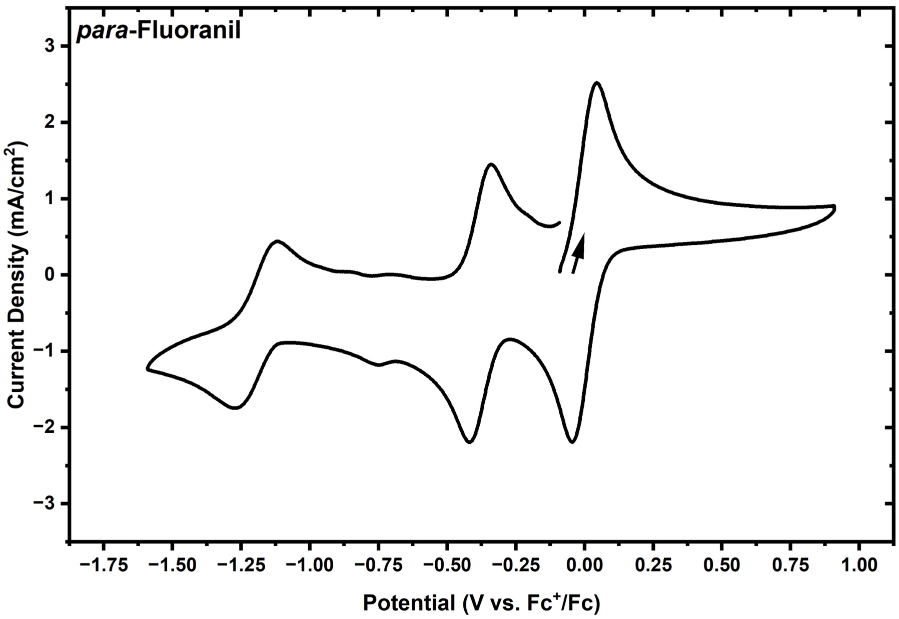

Figure S17. Cyclic voltammogram for *para*-fluoranil in MeCN solution with 0.1 M [(*n*-Bu)$_4$N][PF$_6$] as the supporting electrolyte and ferrocene as an internal reference. Reference electrode: 0.01 M Ag/Ag(NO$_3$) with 0.1 M [(*n*-Bu)$_4$N][PF$_6$] in MeCN with a CoralPor™ frit and Ag wire, working electrode: glassy carbon, counter electrode: Pt wire, scan rate: 0.5 V/s

Table S3. Redox potentials for *ortho*-benzoquinone derivatives in the presence of 1, 2 or 4 equiv of HOPiv.

**Benzoquinone**	**$E_{1/2}$ (V vs. Fc^+/Fc) 1 equiv HOPiv**	**$E_{1/2}$ (V vs. Fc^+/Fc) 2 equiv HOPiv**	**$E_{1/2}$ (V vs. Fc^+/Fc) 4 equiv HOPiv**
9,10-Phenanthrene Dione	-0.813	-0.795	-0.775
1,2-Naphthoquinone	-0.734	-0.709	-0.682
3,5-di-*tert*-butyl-*ortho*-benzoquinone	-0.597	-0.566	-0.563
ortho-chloranil	-0.269	-0.268	-0.255

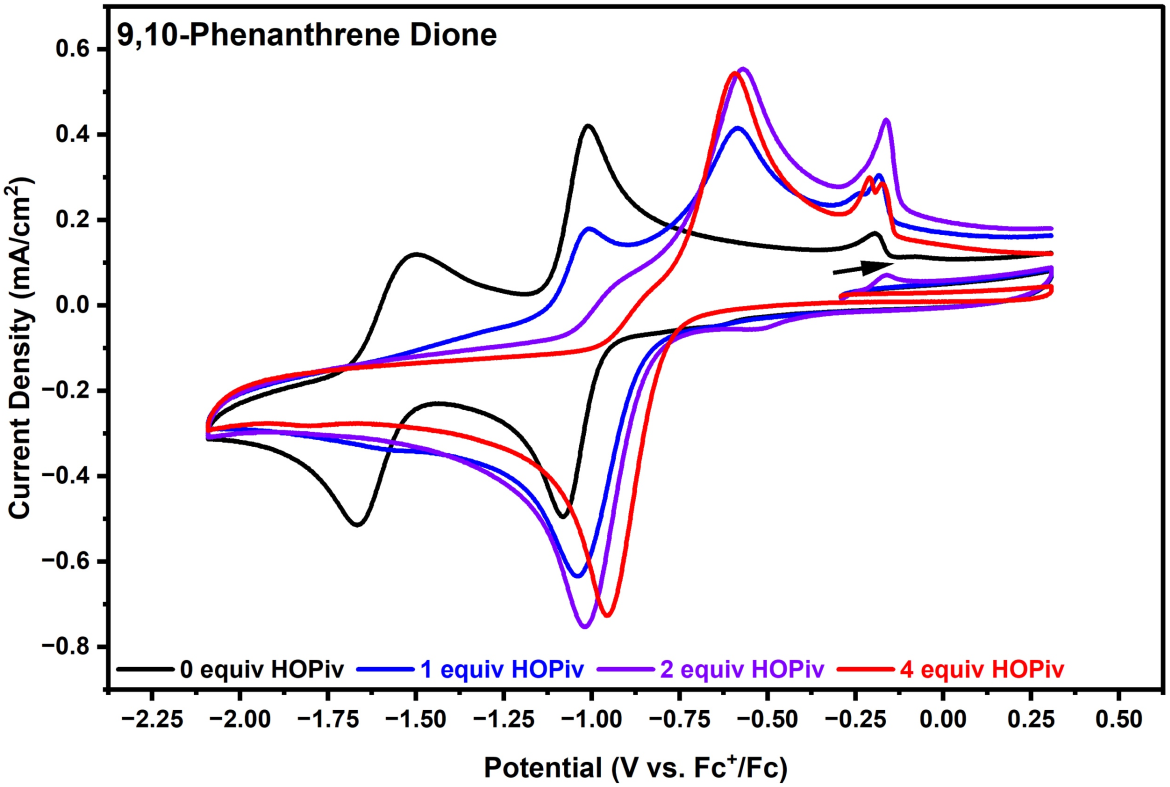

Figure S18. Cyclic voltammograms of 9,10-phenanthrene dione as a function of HOPiv loading in MeCN solution with 0.1 M $[(n\text{-Bu})_4N][PF_6]$ as the supporting electrolyte. Reference electrode: 0.01 M $Ag/Ag(NO_3)$ with 0.1 M $[(n\text{-Bu})_4N][PF_6]$ in MeCN with a CoralPor™ frit and Ag wire, working electrode: glassy carbon, counter electrode: Pt wire, scan rate: 0.2 V/s. Ferrocene was not used as an internal reference in this experiment and its $E_{1/2}$ was measured in a separate scan on the same day, which was found to be 0.092 V vs $Ag/AgNO_3$.

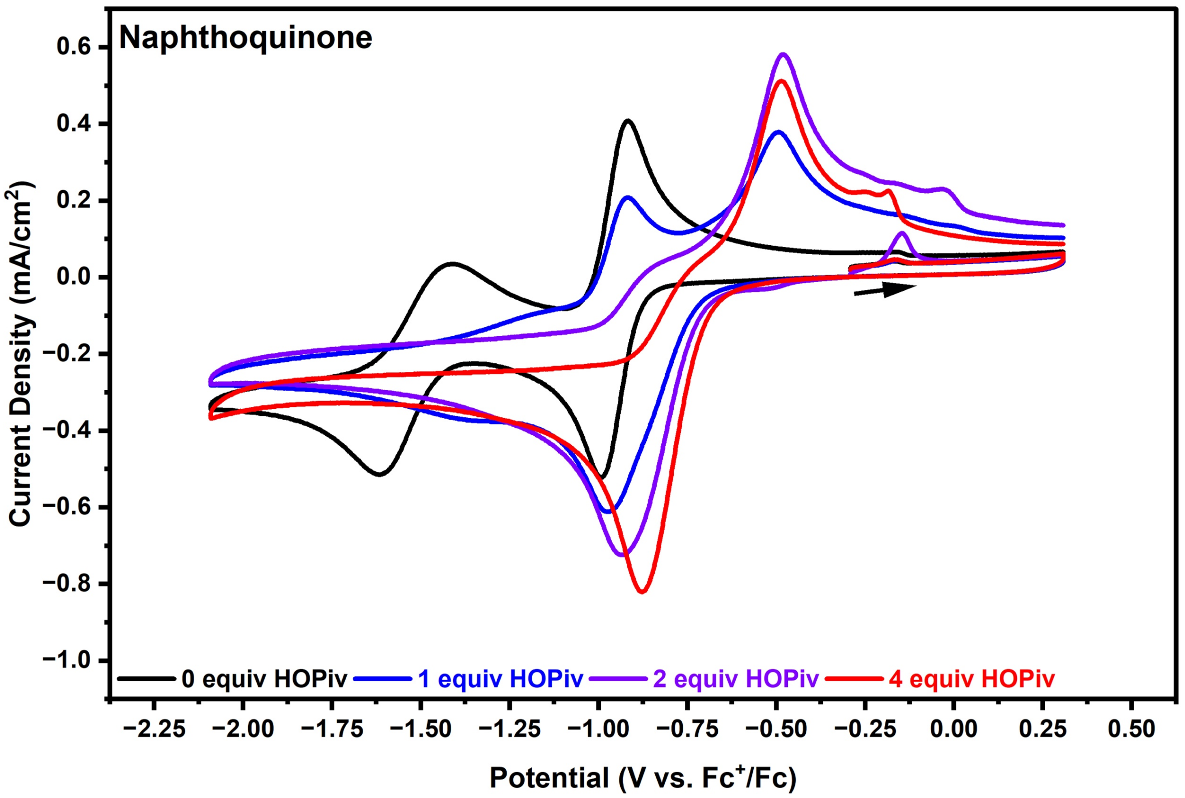

Figure S19. Cyclic voltammograms of Naphthoquinone as a function of HOPiv loading in MeCN solution with 0.1 M [(*n*-Bu)$_4$N][PF$_6$] as the supporting electrolyte. Reference electrode: 0.01 M Ag/Ag(NO$_3$) with 0.1 M [(*n*-Bu)$_4$N][PF$_6$] in MeCN with a CoralPor™ frit and Ag wire, working electrode: glassy carbon, counter electrode: Pt wire, scan rate: 0.2 V/s. Ferrocene was not used as an internal reference in this experiment and its $E_{1/2}$ was measured in a separate scan on the same day, which was found to be 0.092 V vs Ag/AgNO$_3$.

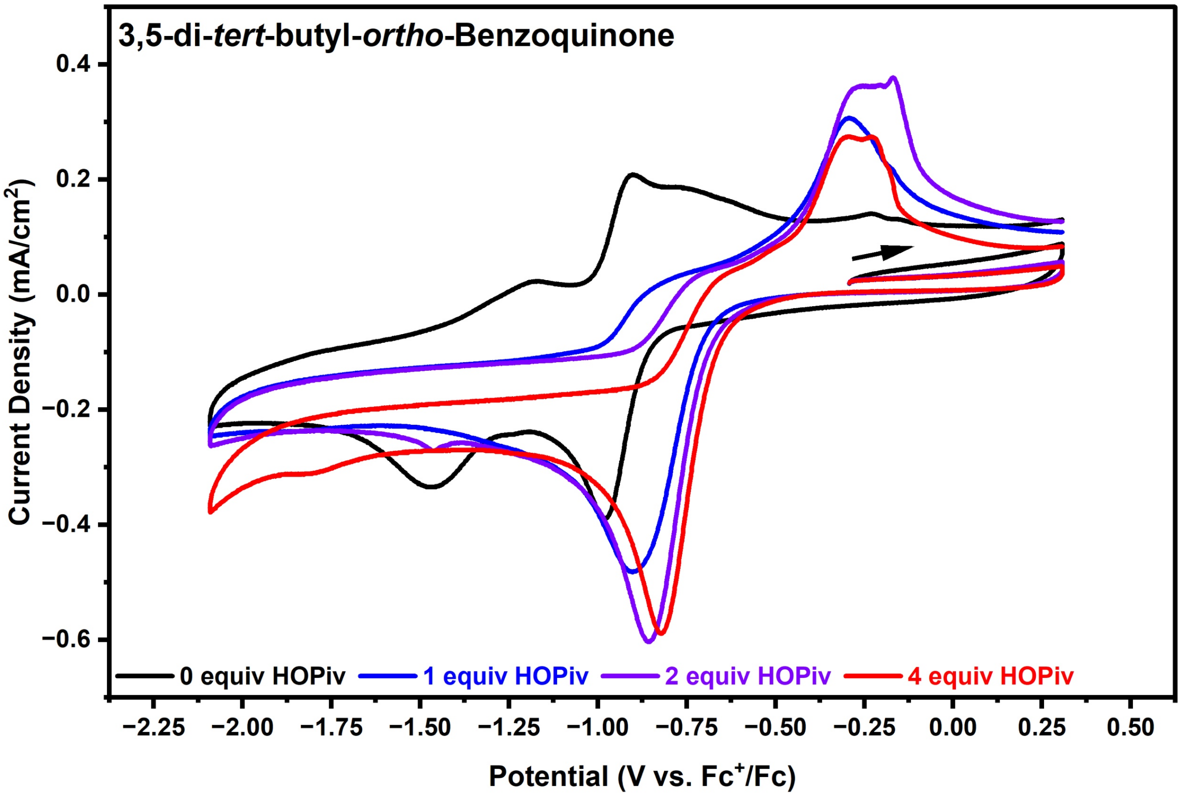

Figure S20. Cyclic voltammograms of 3,5-di-*tert*-butyl-*ortho*-benzoquinone as a function of HOPiv loading in MeCN solution with 0.1 M [(*n*-Bu)$_4$N][PF_6] as the supporting electrolyte. Reference electrode: 0.01 M Ag/Ag(NO_3) with 0.1 M [(*n*-Bu)$_4$N][PF_6] in MeCN with a CoralPor™ frit and Ag wire, working electrode: glassy carbon, counter electrode: Pt wire, scan rate: 0.2 V/s. Ferrocene was not used as an internal reference in this experiment and its $E_{1/2}$ was measured in a separate scan on the same day, which was found to be 0.092 V vs Ag/$AgNO_3$.

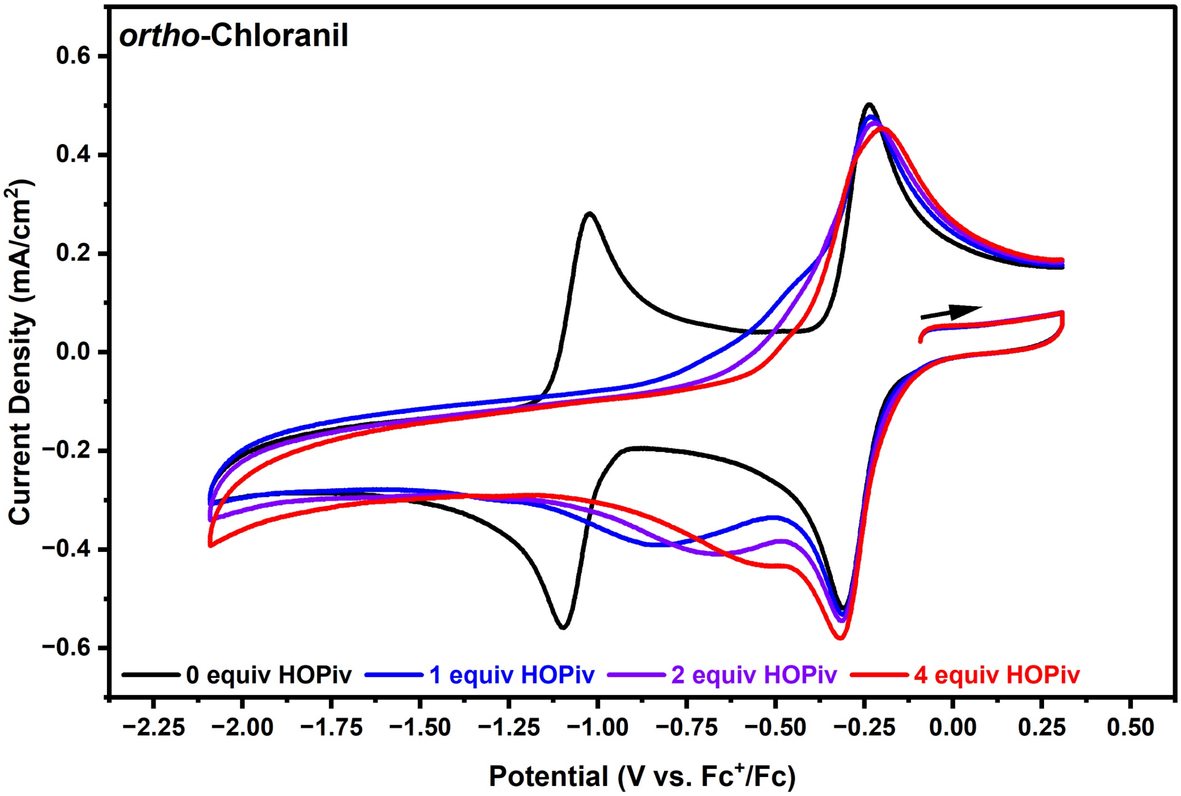

Figure S21. Cyclic voltammograms of *ortho*-chloranil as a function of HOPiv loading in MeCN solution with 0.1 M [(*n*-Bu)$_4$N][PF_6] as the supporting electrolyte. Reference electrode: 0.01 M Ag/Ag(NO_3) with 0.1 M [(*n*-Bu)$_4$N][PF_6] in MeCN with a CoralPor™ frit and Ag wire, working electrode: glassy carbon, counter electrode: Pt wire, scan rate: 0.2 V/s. Ferrocene was not used as an internal reference in this experiment and its $E_{1/2}$ was measured in a separate scan on the same day, which was found to be 0.092 V vs Ag/$AgNO_3$.

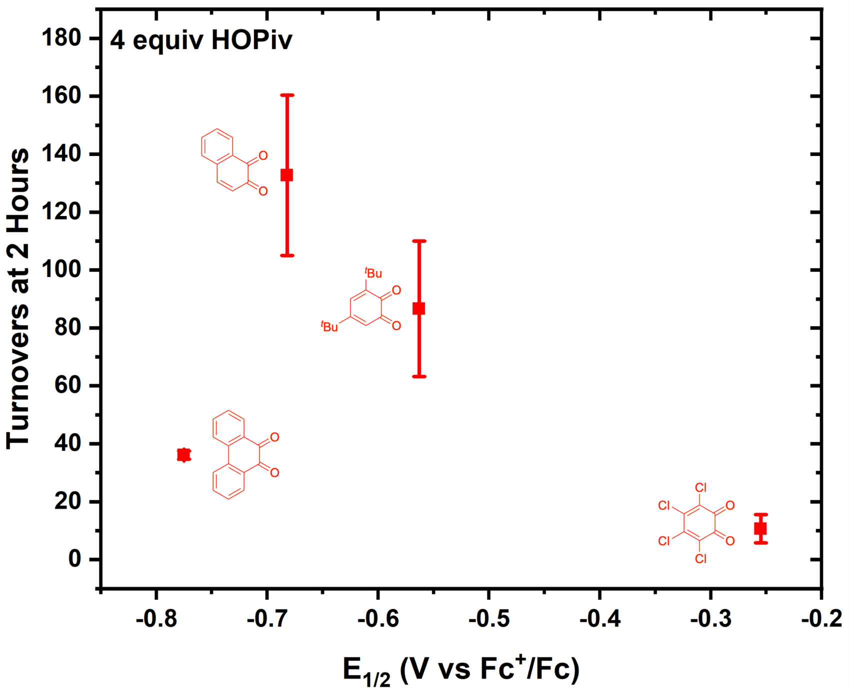

Figure S22. Benzene ethenylation turnover frequency (represented by TOs of styrene measured after 2 hours) versus $E_{1/2}$ for the single redox event of *ortho*-benzoquinone derivatives observed in the presence of 4 equiv of HOPiv. Cyclic voltammograms were recorded in degassed MeCN with 100 mM [*N*-Bu_4][PF_6] (*N*-Bu_4 = tetrabutylammonium) as the supporting electrolyte, and reduction potentials are referenced to ferrocene, which was used as an internal standard. Working electrode: glassy carbon; counter electrode: Pt wire; reference electrode: Ag/$AgNO_3$. Each data point for styrene turnovers at 2 hours represents the average of a minimum of three independent experiments and the error bars represent the standard deviation from the multiple experiments.

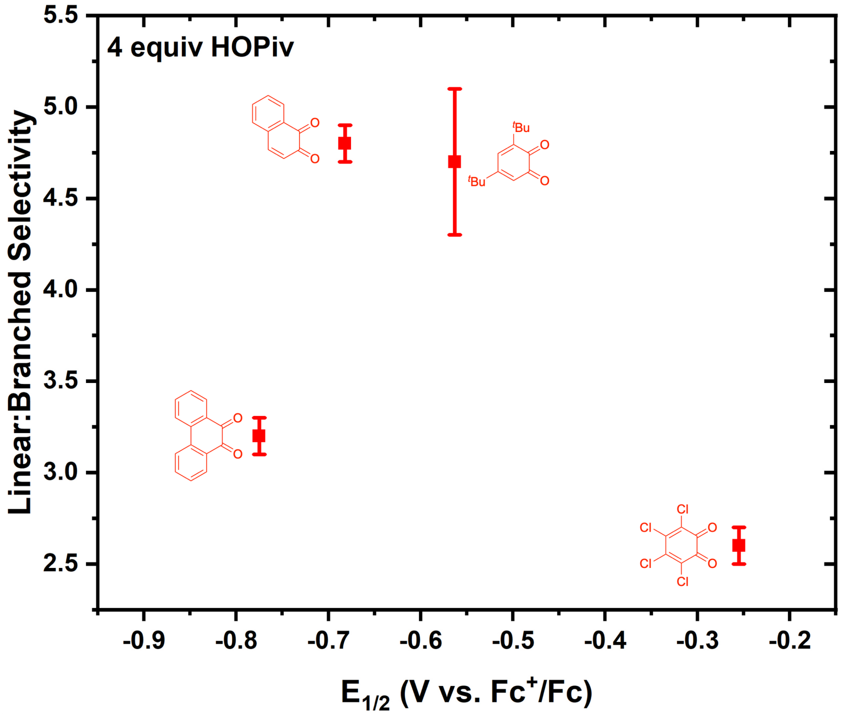

Figure S23. Linear:branched selectivity for benzene propenylation as a function of $E_{1/2}$ for the single redox event of *ortho*-benzoquinone derivatives observed in the presence of 4 equiv of HOPiv. Cyclic voltammograms were recorded in degassed MeCN with 100 mM [*N*-Bu_4][PF_6] (*N*-Bu_4 = tetrabutylammonium) as the supporting electrolyte, and reduction potentials are referenced to ferrocene, which was used as an internal standard. Working electrode: glassy carbon; counter electrode: Pt wire; reference electrode: Ag/$AgNO_3$. Each data point represents the average of a minimum of three independent experiments and the error bars represent the standard deviation from the multiple experiments.

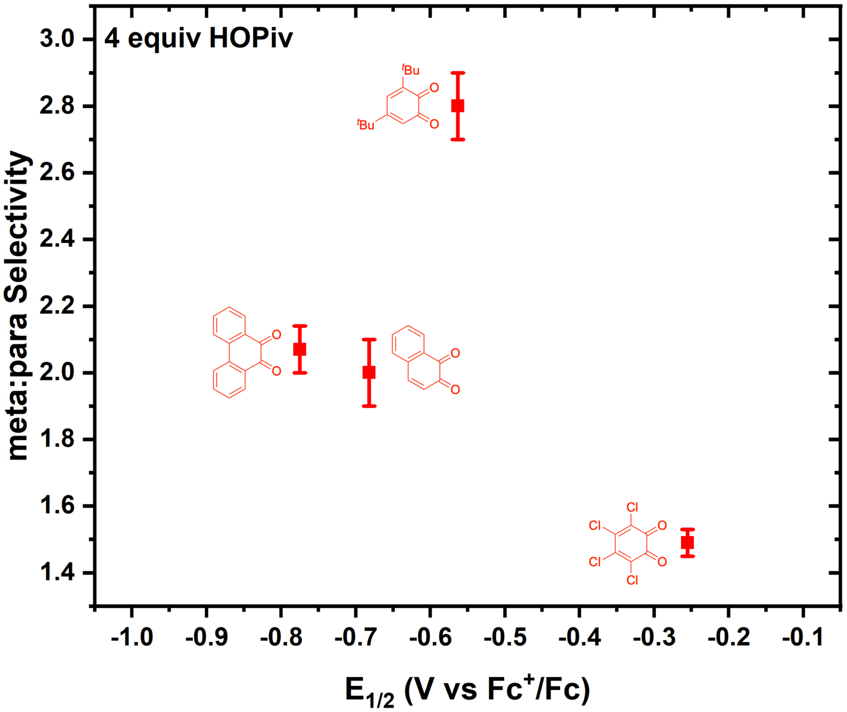

Figure S24. Meta:para selectivity for the ethenylation of *tert*-butylbenzene as a function of $E_{1/2}$ for the single redox event of *ortho*-benzoquinone derivatives observed in the presence of 4 equiv of HOPiv. Cyclic voltammograms were recorded in degassed MeCN with 100 mM [*N*-Bu_4][PF_6] (*N*-Bu_4 = tetrabutylammonium) as the supporting electrolyte, and reduction potentials are referenced to ferrocene, which was used as an internal standard. Working electrode: glassy carbon; counter electrode: Pt wire; reference electrode: Ag/$AgNO_3$. Each data point represents the average of a minimum of three independent experiments and the error bars represent the standard deviation from the multiple experiments.

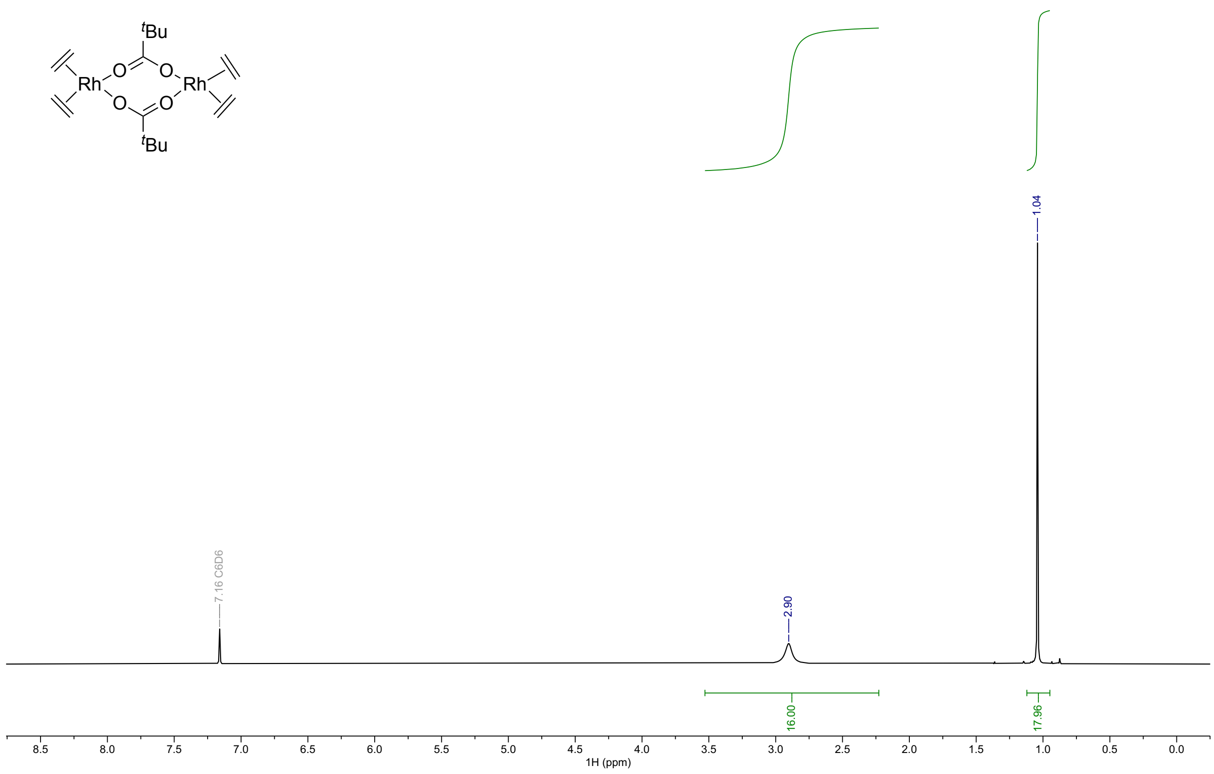

Figure S25. ^{1}H NMR spectrum of $[(\eta^2\text{-}C_2H_4)_2Rh(\mu\text{-}OPiv)]_2$ (600 MHz, C_6D_6).

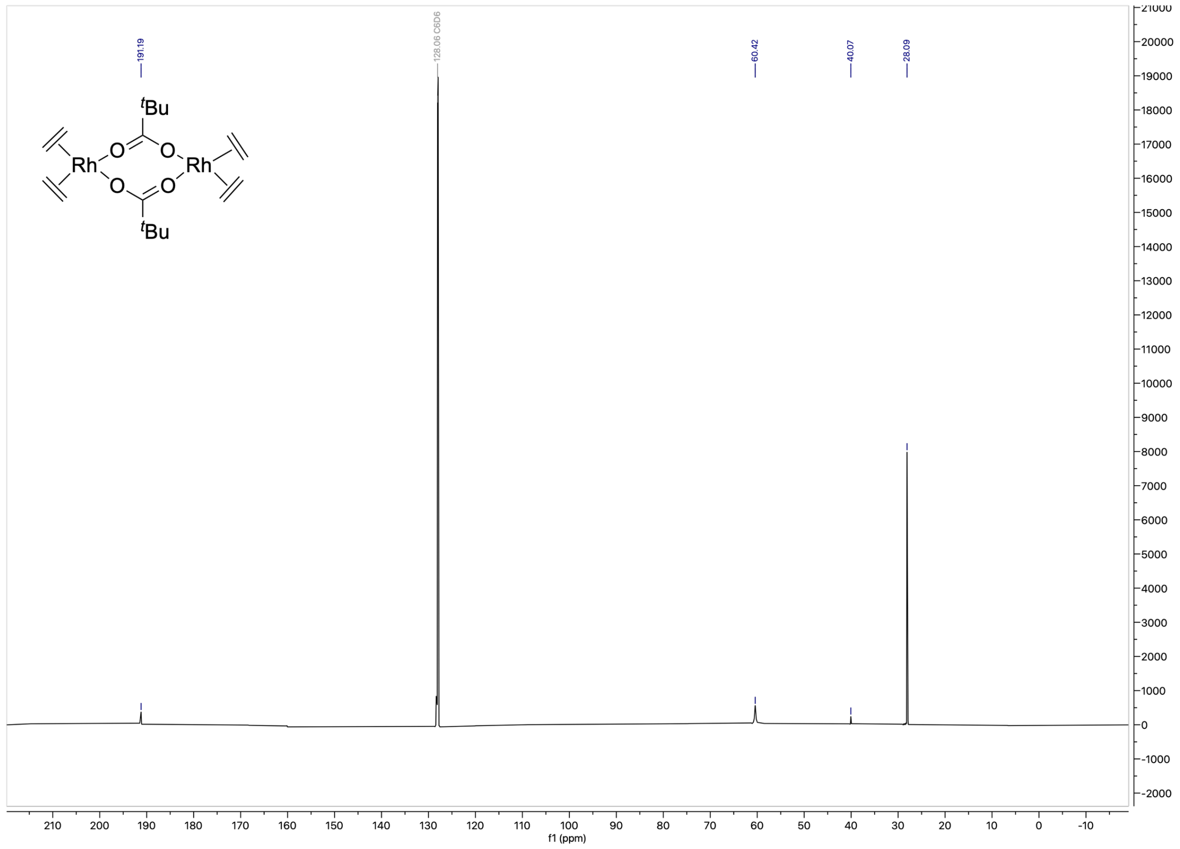

Figure S26. ^{13}C NMR spectrum of $[(\eta^2\text{-}C_2H_4)_2Rh(\mu\text{-}OPiv)]_2$ (200 MHz, C_6D_6).

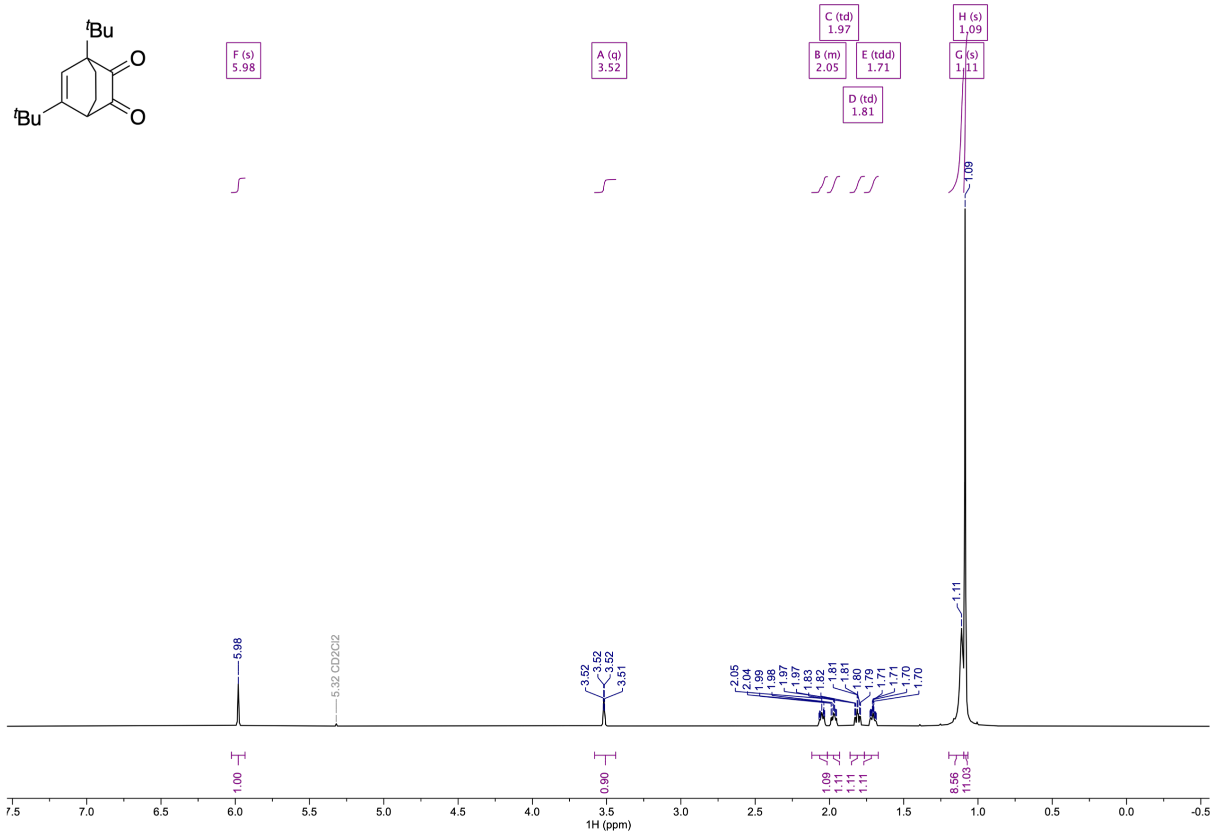

Figure S27. ^{1}H NMR spectrum of 1,5-di-*tert*-butylbicyclo[2.2.2]oct-5-ene-2,3-dione (800 MHz, CD_2Cl_2).

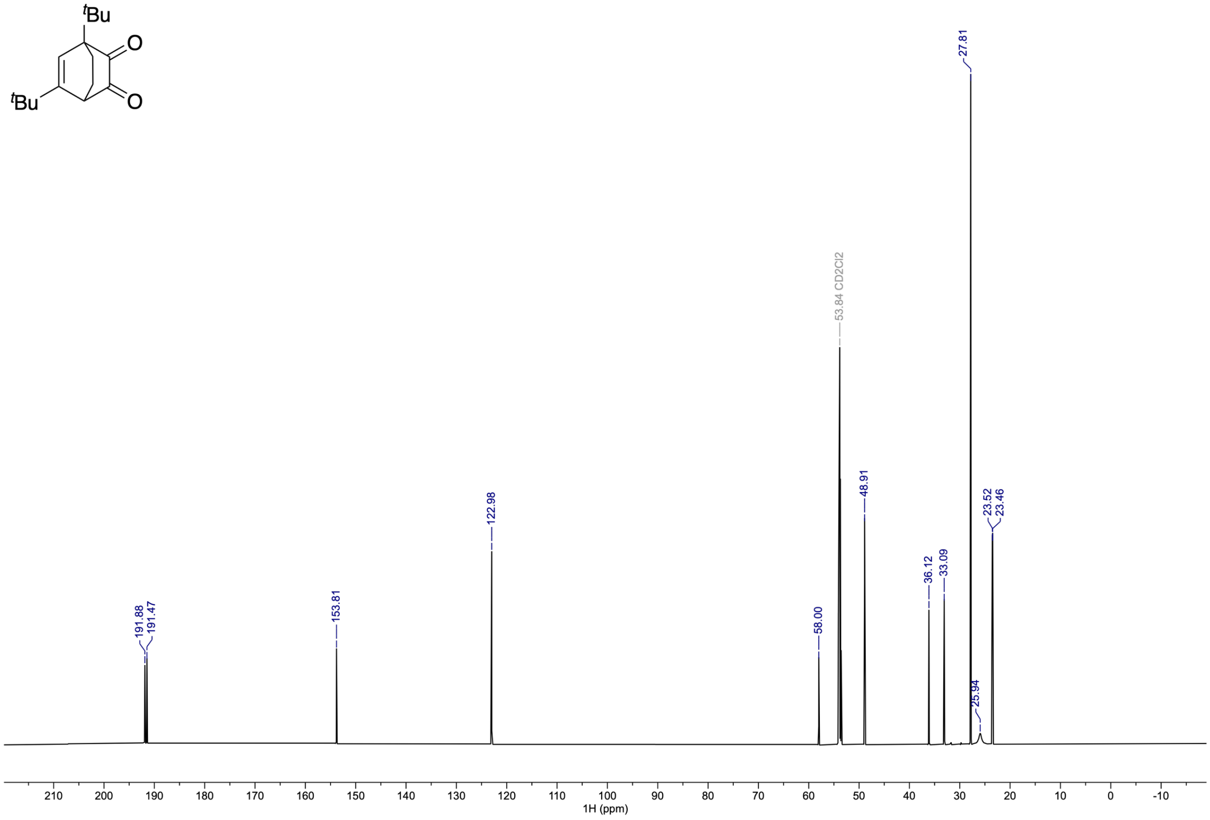

Figure S28. ^{13}C NMR spectrum of 1,5-di-*tert*-butylbicyclo[2.2.2]oct-5-ene-2,3-dione (200 MHz, CD_2Cl_2).

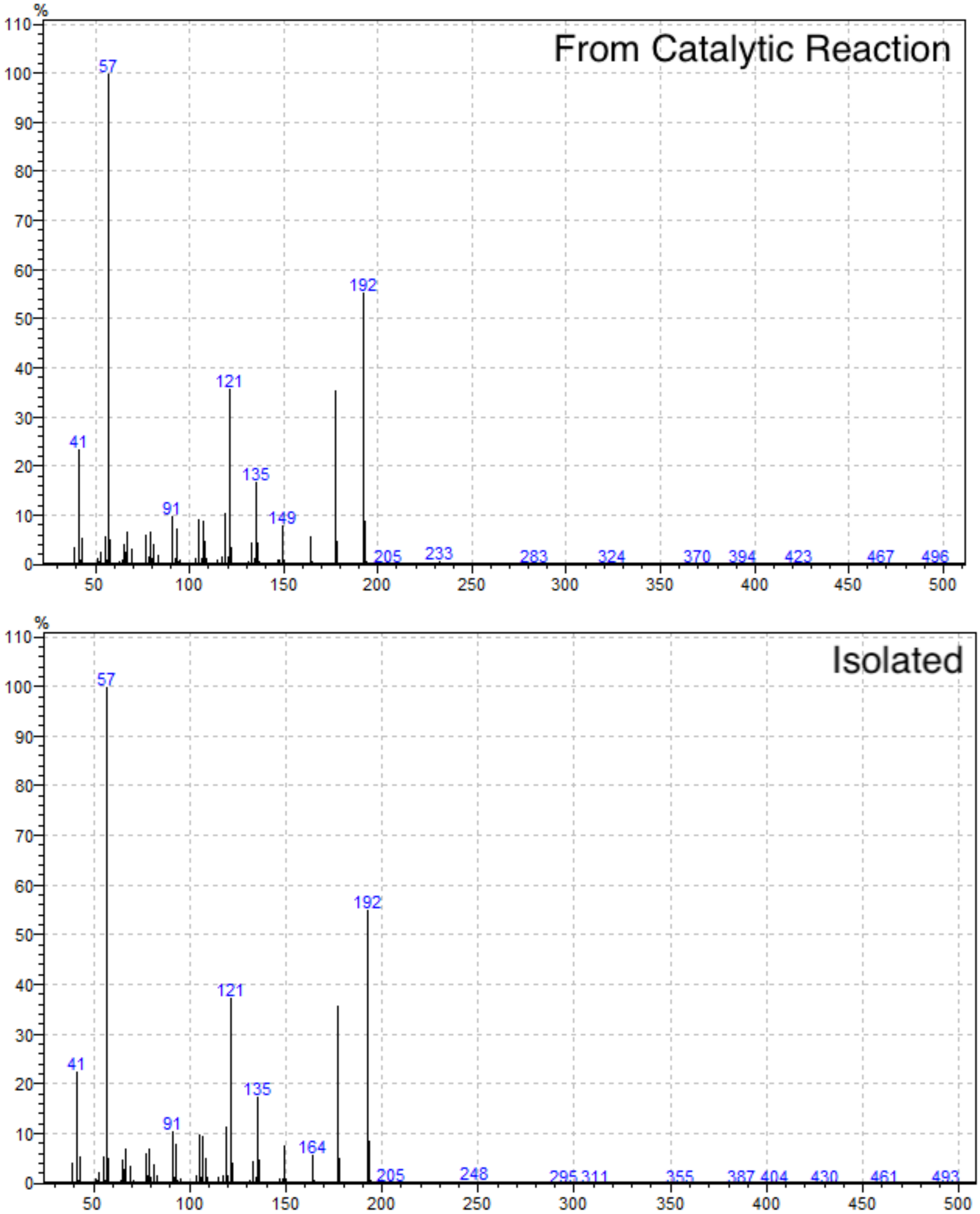

Figure S29. Mass spectra of 1,5-di-*tert*-butylbicyclo[2.2.2]oct-5-ene-2,3-dione obtained from a catalytic reaction and the isolated material.

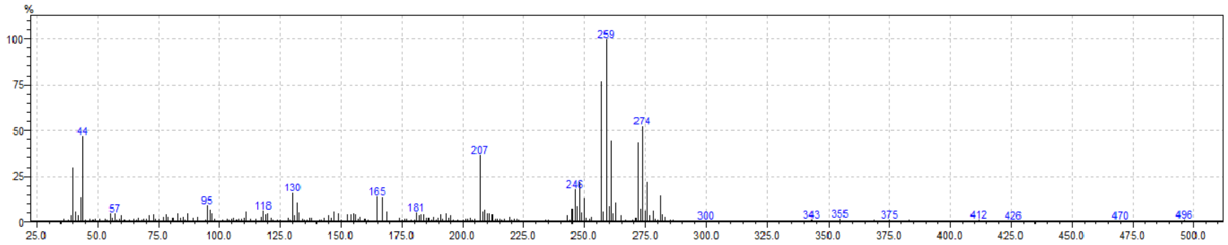

Figure S30. Mass spectrum of the side product observed when using *ortho*-chloranil as the oxidant for benzene ethenylation, which is likely 1,4,5,6-tetrachlorobicyclo[2.2.2]oct-5-ene-2,3-dione.
